# Associations between nucleosome phasing, sequence asymmetry, and tissue-specific expression in a set of inbred Medaka species

**DOI:** 10.1186/s12864-015-2198-5

**Published:** 2015-11-19

**Authors:** Yoichiro Nakatani, Cecilia C. Mello, Shin-ichi Hashimoto, Atsuko Shimada, Ryohei Nakamura, Tatsuya Tsukahara, Wei Qu, Jun Yoshimura, Yutaka Suzuki, Sumio Sugano, Hiroyuki Takeda, Andrew Fire, Shinichi Morishita

**Affiliations:** Department of Computational Biology and Medical Sciences, Graduate School of Frontier Sciences, The University of Tokyo, Kashiwa, 277-0882 Japan; Department of Pathology, School of Medicine, Stanford University, Stanford, CA 94305-5324 USA; Graduate School of Medical Sciences, Kanazawa University, Kanazawa, 920-1192 Japan; Department of Biological Sciences, Graduate School of Science, The University of Tokyo, Tokyo, 113-0033 Japan; Department of Medical Genome Sciences, Graduate School of Frontier Sciences, The University of Tokyo, Tokyo, 108-8639 Japan; Departments of Pathology and Genetics, School of Medicine, Stanford University, Stanford, CA 94305-5324 USA

## Abstract

**Background:**

Transcription start sites (TSSs) with pronounced and phased nucleosome arrays downstream and nucleosome-depleted regions upstream of TSSs are observed in various species.

**Results:**

We have characterized sequence variation and expression properties of this set of TSSs (which we call “Nucleocyclic TSSs”) using germline and somatic cells of three medaka (*Oryzias latipes*) inbred isolates from different locations. We found nucleocyclic TSSs in medaka to be associated with higher gene expression and characterized by a clear boundary in sequence composition with potentially-nucleosome-destabilizing A/T-enrichment upstream (*p* < 10^−60^) and nucleosome- accommodating C/G-enrichment downstream (*p* < 10^−40^) that was highly conserved from an ancestor. A substantial genetic distance between the strains facilitated the in-depth analysis of patterns of fixed mutations, revealing a localization-specific equilibrium between the rates of distinct mutation categories that would serve to maintain the conserved sequence anisotropy around TSSs. Downstream of nucleocyclic TSSs, C to T, T to C, and other mutation rates on the sense strand increased around first nucleosome dyads and decreased around first linkers, which contrasted with genomewide mutational patterns around nucleosomes (*p* < 5 %). C to T rates are higher than G to A rates around nucleosome associated with germline nucleocyclic TSS sites (*p* < 5 %), potentially due to the asymmetric effect of transcription-coupled repair.

**Conclusions:**

Our results demonstrate an atypical evolutionary process surrounding nucleocyclic TSSs.

**Electronic supplementary material:**

The online version of this article (doi:10.1186/s12864-015-2198-5) contains supplementary material, which is available to authorized users.

## Background

Nucleosomes constitute the basic building blocks of DNA chromatin structure and play a pivotal role in regulating genes. Genome-wide examinations of nucleosome positioning in model organisms [1–10], as well as in humans [11–16], have uncovered a variety of associations among nucleosome positioning and spacing probabilities, underlying DNA sequence composition, histone modification, TSS distribution, gene expression, and rates of mutagenesis and repair in various tissue types in different organisms.

Recent studies have identified arrays of positioned and phased nucleosomes downstream of TSSs and eviction of nucleosomes upstream as permissive features associated with access of transcription factors to the DNA [3, 7, 9, 12, 17, 18]. Weak and strong phased nucleosome arrays respectively associate with narrow and broad distributions of TSSs upstream of individual genes in budding yeast [19, 20], human CD4^+^ T cells and fruitfly cells [21]. Nucleosome spacing downstream of TSSs is associated with differential expression levels and histone modification [22, 23]; as an example in a vertebrate system, crowding of TSS-downstream nucleosomes is associated with higher expression in human white blood cells, with average spacing decreased at high expression levels from 195 bp to 190 bp in granulocytes and from 205 bp to 195 bp in CD4^+^ T cells [12]. Transcription is just one of the forces that may influence and be influenced by nucleosome positioning. Additional contributory factors include histone protein modifications [11], distribution of histone sequence variants, a variety of chromatin remodeling complexes [24], specific transcription factors, and regulatory RNAs.

Underlying all of these are effects of DNA sequence composition including ~10 bp periodicities of AT-dinucleotide and CG-dinucleotide frequencies around nucleosome dyads [3, 5, 8, 18, 25, 26], a higher poly-A incidence in nucleosome depleted region upstream of TSSs [7, 25, 27], a higher GC/GG/CC incidence surrounding nucleosome dyads and a higher AA/TT incidence around linker regions [12, 27]. Genome-wide nucleosome positioning is also relevant to rates of genetic variations that may contribute to sequence composition biases around nucleosomes [10, 13, 14, 26, 28–30]. Around nucleosome dyads in the entire human genome, somatic mutations are suppressed in cancer tissues [28], and *de novo* germline mutations are less observed [29]. In budding yeast and medaka, spontaneous variants (e.g., C to T, G to T, and A to T) in nucleosome core regions are decreased, potentially contributing to higher GC incidences around nucleosome dyads [10]. In the fruitfly genome, ~10 bperiodicities of AT-dinucleotide and CG-dinucleotide frequencies are evolutionarily conserved [26]. In the human genome, Prendergast and Semple, through an analysis of inter-species divergence and intra-species polymorphism, observed higher rates of W (A/T) to S (C/G) mutations in core regions around nucleosome dyads and higher rates of S (C/G) to W (A/T) changes at linker regions, suggesting that genetic variation might be intrinsically biased to maintain high GC incidences around nucleosome dyads and lower GC incidences at linker regions [14].

Around TSSs, chromatin-associated periodicity in genetic variation downstream of TSSs has been reported in germline-like, early embryonic cells of medaka-fish [9] and in various human cells [13]; however, the relationships between nucleosome positioning, underlying sequence composition, and directions of mutations around TSSs are largely unexplored, leaving unresolved some fundamental questions surrounding the mechanisms by which species maintain persistent features of chromatin organization while showing substantial sequence variability over evolutionary time. The medaka fish (*Oryzias latipes*) provides an ideal genomic vertebrate resource to study these issues, in part due to the ready availability of strains with substantial sequence divergence but continued cross-fertility. In particular, the genomes of two inbred strains, Hd-rR and HNI, show high genetic variation of ~3.42 % (SNP rate [31]), while the genome of an outgroup inbred strain HSOK to Hd-rR and HNI can be used to allow a strong inference of the ancestral states at a large number of sites (see the phylogenetic tree of the three strains in Additional file 1: Figure S1a). The availability of germline tissues, as the site of any sequence changes that become fixed in a population, provides a particularly relevant context for examining the interactions between genome architecture and evolution. Comparative studies of genomic and epigenomic organization, well served in the medaka model system, are amongst the best poised approaches to address the persistent and/or dynamic relationships between sequences and chromatin structure over evolutionary time. We observed atypical evolution around TSSs with phased downstream nucleosomes (which we call “Nucleocyclic TSSs”) that differed from the characteristics of genome-wide nucleosome positioning.

## Results and discussion

### Transcription start sites: Experimental approach, normalization, and definition

**Fig. 1:**
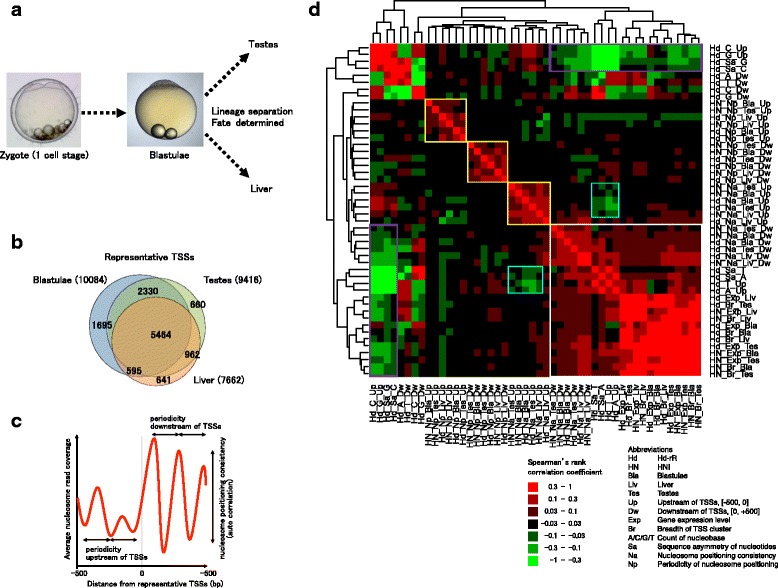
**a**. Focal tissue types during embryogenesis and lineage separation. **b**. Venn diagram of the three sets of representative TSSs that were detected in the respective tissue types in our sage data: blastulae, testes, and liver. Numbers of TSSs are labeled with individual subsets. **c**. Schematic showing the periodicity of nucleosomes upstream or downstream of a representative TSS using the autocorrelation analysis that quantifies the nucleosome positioning consistency (Methods). **d**. Spearman’s rank correlation coefficient matrix for the data deriving from the Hd-rR (Hd) and HNI (HN) strains. The individual values can be found in Additional file 12: Table S1. The gene expression (Exp), breadth of TSS cluster (Br), nucleosome periodicity (Np) and nucleosome positioning consistency (measured by autocorrelation) were monitored in blastula (Bla), liver (Liv), and testes (Tes). Single nucleotide count of each nucleobase (A, C, G, and T), nucleosome periodicity (Np), and nucleosome consistency (Na) were separately calculated in upstream (Up) and downstream (Dw) TSS regions. The difference of nucleobase counts upstream and downstream of TSSs is called the sequence asymmetry of nucleotides (Sa). To exemplify the nomenclature used, Hd_A_Dw denotes the incidence in the Hd-rR strain of nucleotide A downstream of TSSs. We also report other sequence composition features, A+T (A or T), C+G, AA+TT, and CC+GG, and their sequence asymmetry values; however, we note that these are not independent of single base composition features (and we thus did not use them to avoid redundancy) (see Additional file 1: Figure S1c). We considered 1128 combinations of 48 parameters as candidate hypotheses, with some of the comparisons showing positive or negative association due to technical aspects of the quantitation (*e.g.*, breadth and expression level in a given tissue are associated based on the increased sampling for high expression genes) while others showed positive or negative association due to their intrinsic definitions (*e.g*., T content upstream and G content upstream are expectedly negatively associated). A Bonferroni correction, a typical multiple hypothesis testing method, is valuable to rigorously test each hypothesis. A significance level of 5 %/1128 (~4.4 x 10^−5^) would require *r* (Spearman’s rank correlation coefficient) of |*r*|> 0.03, achieving a *p*-value < 10^−43^ when |*r*|> 0.1. The white box shows a high correlation among nucleosome consistency downstream of TSSs in each tissue type (Hd/HN_Na_Bla/Liv/Tes_Dw), sequence asymmetry (Hd_Sa_A/T), A/T single nucleotide counts upstream of TSSs (Hd_A/T_Up), gene expression level (Hd/HN_Exp_Bla/Liv/Tes) and breadth of TSS cluster (Hd/HN_Br_Bla/Liv/Tes). The three yellow boxes imply high correlations among periodicity of nucleosome positioning upstream (or, downstream) of TSSs in the three tissues of the two strains, and a high correlation among consistency of nucleosome positioning upstream of TSSs. The cyan boxes mean that nucleosome positioning consistency upstream of TSSs are negatively correlated with sequence asymmetry and count of A and T. The two purple boxes are similar to the white box except for the negative correlation of C/G single nucleotide counts upstream of TSSs (Hd_C/G_Up) with the other parameters in the white box

To characterize transcription start sites, we collected 36-nucleotide, 5′-end mRNA tags from testes, blastulae, and liver (Fig. 1a) from two inbred medaka strains, Hd-rR and HNI, using a 5′SAGE method [32]. A total of ~10 million reads was collected from each tissue type, of which 71 % could be aligned to unique positions in their respective genomes, Hd-rR or HNI (Table 1). Each TSS was then associated with the normalized number of reads mapped to the location, providing a surrogate quantification of the expression level of the TSS. Starting with the working approximation that each cell expresses on the order of 300,000 mRNA molecules (an estimate from human cells [33]), our datasets of ~9 million tags would yield roughly 30 tags for a single-copy-per-cell mRNA. Such a normalization is by nature arbitrary, with the application here being solely to provide an order of magnitude context in discussing comparisons between samples (see Methods).

**Table 1:** Number of 5′-end reads collected from three tissues of the two medaka inbred strains according to the 5′-SAGE method. Alignment to the genome was performed using ELAND using default parameters

	Total reads	Mapped reads	Uniquely mapped reads	Ratio (%)
Hd-rR blastulae	8,841,684	6,461,567	5,486,904	62.1 %
Hd-rR testes	12,007,331	7,422,679	6,682,875	55.7 %
Hd-rR liver	8,284,838	6,206,848	5,766,607	69.6 %
HNI blastulae	11,404,141	10,629,048	8,385,917	73.5 %
HNI testes	10,419,993	9,442,123	8,585,879	82.4 %
HNI liver	10,856,232	9,844,800	9,051,809	83.4 %
Total	61,814,219	50,007,065	43,959,991	71.1 %

While some genes possess a single TSS, most genes have clusters of TSSs in their upstream region. To proceed with this study, we needed a list of TSS sites for many genes, but wanted to avoid assumptions that this list would be unique or comprehensive. We thus defined the representative TSS for each gene as the position associated with the maximum sequence tag count (see Methods). Activity patterns of TSSs vary among tissues, thus it was not surprising that some TSSs were observed only in one or two tissues from a given strain. Figure 1b shows a Venn diagram of the three sets of representative TSSs that are expressed in the three individual tissue types of the Hd-rR strain. A total of 12,347 representative TSSs were observed from all three tissues, and 5464 were common in the three, but many TSSs were only observed in one or two tissues.

We defined one-to-one correspondence between TSS clusters in the Hd-rR and HNI genomes by carefully selecting reciprocally best pairs of alignments (Methods). A TSS cluster in Hd-rR may not have a counterpart in HNI because the quality of the HNI genome assembled from short reads was not sufficiently high (Methods). Nevertheless, 12,347 pairs of TSS clusters in the two strains were available. Thus, we measured the distance between pairs of corresponding representative TSSs in the Hd-rR and HNI strains. The distance distribution in Additional file 1: Figure S1b shows that the distance is smaller than or equal to 0, 10, 50, and 100 bp for 18.5 %, 53.9 %, 84.1 %, and 89.8 % of 12,347 TSS pairs, indicating that representative TSSs are likely to be positionally conserved between the two strains.

### Periodicity and consistency of nucleosome positioning

We collected nucleosome positioning information for the three tissues from each of the two strains by processing 25-nt single-end reads from nucleosome cores (Table 2; procedure described in Methods). Nucleosomes patterns are known to reflect transcriptional patterns in several systems; in particular, nucleosomes are positioned and phased downstream of many TSSs while a lack of nucleosome reads over active promoters has evidenced their lability or eviction from promoters [3, 7, 9, 12, 17]. Although mainly seen downstream, phased nucleosomes are also observed upstream of some TSSs, potentially evidencing bidirectional promoter usage [22]. We measured the periodicity and consistency of nucleosome positioning within the two separate 500-bp regions downstream and upstream of a representative TSS by using autocorrelation analysis (Fig. 1c, Methods). To minimize the effects of neighboring TSS on nucleosome positioning and sequence composition, representative TSSs were selected so that each TSS was >500 bp distance apart from its neighbors (Methods).

**Table 2:** Number of single-end reads of mono-nucleosome cores isolated from MNase-digested DNA samples. Alignment to the genome was performed using ELAND using default parameters. The genome coverage was estimated as the number of uniquely mapped reads multiplied by the length of mono-nucleosomes (147 bp) divided by the sequenced medaka genome size (700 M bp). Since single-end short reads of length 25 nt were collected from MNase fragments, the accurate length of individual fragment could not be estimated. To have an approximate picture of the distribution of fragments obtained from MNase digestion, 32 arbitrary fragments were inserted into a standard plasmid vector and sequenced using Sanger sequencing. Of 24 sequences that could be anchored to unique positions, the average length was 150.2 nt with a standard deviation of 9.3 nt. If we use the actual fragment lengths in place of the ideal length (147 nt), the coverage would increase by ~2 %. The cumulative ratio of nucleotides covered by ≥ *x* (=1,2, …, 30) nucleosome core reads are shown in Additional file 1: Figure S1d, which indicates >50 % of the entire genome is covered by ≥ 30 nucleosome core reads in all the tissue types except for HNI testes (~16 reads)

	Collected reads	Uniquely mapped reads	Ratio of uniquely mapped reads	Genome coverage
Hd-rR blastulae	339,407,788	220,270,276	64.90 %	46.3
Hd-rR testes	389,257,067	245,192,521	62.99 %	51.5
Hd-rR liver	342,386,421	233,904,210	68.32 %	49.1
HNI blastulae	391,519,667	291,165,889	74.37 %	61.1
HNI testes	587,561,947	336,898,284	57.34 %	70.7
HNI liver	467,963,072	343,765,549	73.46 %	72.2
Total	2,518,095,962	1,671,196,729	66.37 %	351.0

### Underlying DNA sequence composition

Within defined regions of the genome, nucleosomes are known to preferentially associate with DNA segments exhibiting high C + G and CC + GG content, with some degree of exclusion from corresponding A + T and AA + TT rich regions [1, 34–37]. Although these underlying characteristics may be one of the sequence features specifying nucleosome positioning, it was nonetheless of interest to test their association with nucleosome positioning signals in the vicinity of TSSs. To evaluate potential associations, we measured the count and asymmetry of each of the mononucleotides and dinucleotides around individual representative TSS comparing a 500 nt window upstream and downstream of each TSS. Because some of these parameters were highly correlated (Additional file 1: Figure S1c), we eliminated the redundancy and used mononucleotide content.

### Features associated with the representative TSSs

With each representative TSS, we tested associations of a set of parameters: gene expression level (in log scale), breadth of the TSS, mononucleotide counts and mononucleotide asymmetry values upstream and downstream of the TSS, and periodicity and consistency of nucleosome positioning upstream and downstream of the TSS (see details in Methods). Figure 1d shows the Spearman’s rank correlation of each parameter to the others, using data from liver, testes, and blastulae from the Hd-rR and HNI strains, providing us with the overall characteristics of the relationships among the parameters. Among nucleosome-positioning parameters, the periodicity upstream and downstream did not highly correlate with the other parameters. The nucleosome positioning consistency upstream of a representative TSS was negatively correlated with A/T nucleotide asymmetry and A/T count upstream of the TSS. In contrast, the nucleosome positioning consistency downstream of a representative TSS was significantly correlated with A/T nucleotide asymmetry and A/T nucleotide count upstream of the TSS, gene expression level, and breadth of the TSS (Fig. 1d, *p* < 5 x 10^−5^).

### A class of promoters with strong nucleosomal periodicity (“nucleocyclic promoters”) show higher gene expression and a clear sequence composition boundary

**Fig. 2:**
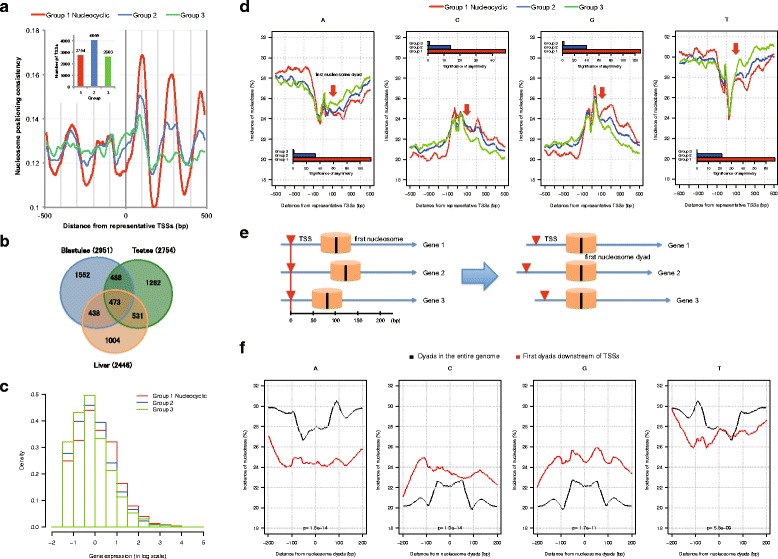
Characteristics of nucleocyclic TSSs in the testes. **a**. We clustered representative TSSs into three groups (denoted by 1, 2, and 3) according to nucleosome consistency (autocorrelation) downstream of the TSSs in testes. A running average over 21-bp window is shown for smoothing of lines. The upper left box shows the number of TSSs in each group. **b**. Venn diagram of nucleocyclic TSSs (Group 1) in blastulae, testes, and liver. **c**. Histogram of probability distribution of gene expression in log scale. The gene expression of the nucleocyclic TSSs in Group 1 is significantly higher than the distribution in Group 2 or 3 (*p* = 1.9 x 10^−17^ by Wilcoxon’s ranksum test). **d**. Average incidences of nucleobase A, T, C and G at positions within 500 bp from representative TSSs in Groups 1, 2 and 3. A running average over 41-bp window is shown. The small histogram in each graph shows the significance of asymmetry of each incidence in individual group (see Methods). A *p*-value is depicted by –log (*p*-value) so that a lower *p*-value is represented by a higher bar to emphasize the higher significance of asymmetry. The difference between the mononucleotide compositions upstream and downstream of TSSs is most pronounced in Group 1. The graphs also indicate that the base incidences around the first nucleosome dyads and linkers significantly differ from those incidences around nucleosomes in the entire genome. **e**. To verify this hypothesis, individual first nucleosome dyads [+50, +150] downstream of nucleocyclic TSSs are identified (see Methods). **f**. Incidences of four bases in the region within 200 bp from nucleosome dyads in the entire genome (black line) and first dyads downstream of TSSs (red line). Lines are smoothed using a running average over 41-bp window. Around nucleosome dyads in the entire genome, the A/T (C/G, respectively) incidences around dyads are smaller (greater) than those around linkers, while this tendency is less pronounced around first dyads downstream of TSSs. Indeed, the difference in the tendency is significant, with *p* < 10^−8^ for any of A, C, G, and T by one-tailed Wilcoxon’s ranksum test (Methods)

We clustered representative TSSs into three groups in terms of their nucleosome positioning consistency downstream of TSSs in the Hd-rR strain (Fig. 2a). Group 1 has the strongest nucleosome positioning, and we called the TSSs in Group 1 “nucleocyclic.” Nucleocyclic TSSs are not common among genes active in the three tissues; many of them were specific to individual tissue types (Fig. 2b), reflecting representative TSSs that have different nucleosome positioning consistency values and expression levels in individual tissue types [12]. We found that the nucleocyclic TSSs had a significantly higher average gene expression level in all three tissue samples (sperm Fig. 2c, *p* < 10^−16^ by one-tailed Wilcoxon’s ranksum test described in Methods; blastulae in Additional file 2: Figure S4b and liver in Additional file 3: Figure S5b; *p* < 10^−4^ and *p* < 10^−6^ respectively). The nucleocyclic TSSs also showed a significantly asymmetric distribution of each mononucleotide around the TSSs (Fig. 2d), average A/T nucleotide incidences upstream of the TSSs were significantly higher than the incidences downstream (*p* < 10^−116^ for A and *p* < 10^−68^ for T by a two sample *z*-test described in Methods).

From these observations, we speculate that the greater A/T nucleotide incidence upstream of nucleocyclic TSSs might repel or destabilize nucleosomes, with the C/G-rich character downstream accommodating arrays of well-positioned nucleosomes (a schematic version of this model is diagrammed in Additional file 4: Figure S3). The comparison between sequence composition around first downstream nucleosomes and nucleosomes not selected as being in transcribed regions suggests a unique local characteristic around the first downstream nucleosome with mononucleotide incidences varying little between the dyad and linker (Fig. 2f, Additional file 2: Figure S4d, and Additional file 3: Figure S5d). As with yeast [27] and human [12], a genomewide analysis of nucleosome dyad and linker regions shows an enrichment in A/T content around the latter. For first nucleosome dyads downstream of individual nucleocyclic TSSs in medaka (Fig. 2e), we observe that the mononucleotide incidence difference is significantly smaller than the difference for all (genome-wide) nucleosomes (*p* < 10^−8^, Fig. 2f).

The combinatorial effect of the A/T and C/G biases around nucleocyclic TSSs, the atypical sequence composition around the first downstream nucleosome, combined with processes such as RNA PolII stalling, could then induce the stable positioning of downstream nucleosomes, thereby potentially anchoring TSSs and permitting a high level of transcription initiation at specific sites. Considering the prevalence of transcriptional initiation at nucleocyclic TSSs, we raise the possibility that nucleocyclic TSSs may be more positionally conserved between the two strains than non-nucleocyclic TSSs. Indeed, we confirmed this tendency in testes (*p* < 10^−9^), in blastulae (*p* < 10^−10^), and in liver (*p* < 10^−32^) using one-tailed Wilcoxon’s ranksum test (Methods).

### Atypical evolution around nucleocyclic TSSs

**Fig. 3:**
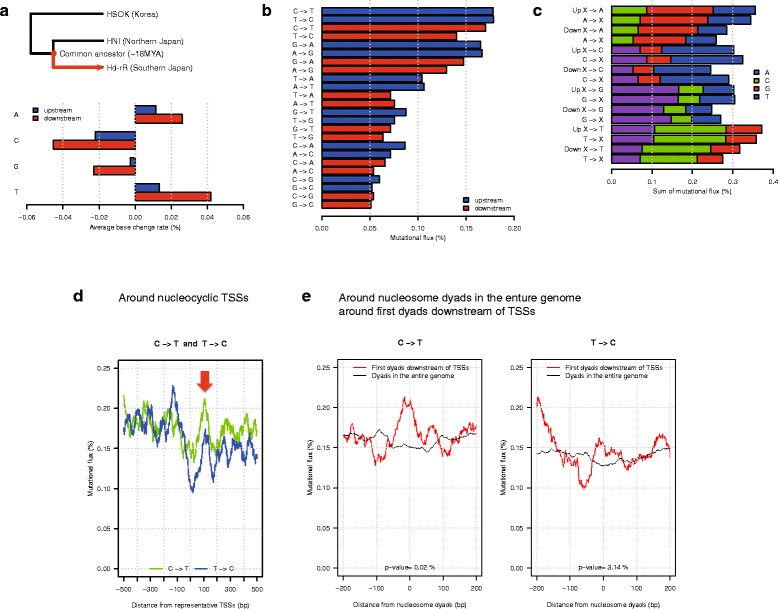
Atypical evolution around nucleocyclic TSSs in testes. **a**. Average mononucleotide incidence changes from the common ancestor of Hd-rR and HNI to the current Hd-rR genome upstream and downstream of nucleocyclic TSSs (positive values: net increase, negative values: net decrease). The A/T incidence increases and C/G incidence decreases are quite small (−0.05 % ~ 0.05 %). The mononucleotide compositions are almost conserved from the ancestor to Hd-rR genome. The mononucleotide incidence changes are more pronounced in downstream regions than in upstream regions. **b**. The histogram shows “mutational flux”, average rates of base change from ancestral base X to current Hd-rR base Y upstream/downstream of nucleocyclic TSSs. **c**. For each mononucleotide Z (A, C, G, or T), the histogram shows the sum of mutational flux, average mutation rates X to Z (the flow into Z), where X is a mononucleotide other than Z, and the sum of Z to X (the flow out from Z) upstream (denoted “Up” in the figure)/downstream (Down) of nucleocyclic TSSs. **d**. Rates of average base change at positions around nucleocyclic TSSs (see all changes in Additional file 9: Figure S8a). A running average over 41-bp window is shown in Fig. 3d-e. The red arrow show the approximate positions of the first nucleosome dyads downstream of the nucleocyclic TSSs, indicating that C to T, and T to C rates have peaks at the first dyads and valleys at the first linkers (see also Additional file 7: Figure S10d for the liver case). **e**. We analyzed the apparent the nucleosome position-related mutational bias through analysis of mutational fluxes following alignment of first nucleosomes. After identifying the first nucleosome dyads downstream of TSSs according to the method described in Fig. 2e mutational fluxes are graphed in relation to the nucleosome dyad. First nucleosomes (red lines) and genomewide nucleosomes (black lines) show a significantly opposite trend around the dyads in the entire genome; namely, *p* = 0.02 % and 3.14 % for C to T and T to C, respectively by one-tailed Wilcoxon’s ranksum test

The conservation of the clear sequence composition boundary around nucleocyclic TSSs led us to examine whether specific properties of the mutagenic landscape around these TSSs might be involved in the maintenance of the boundary. The genomes of two medaka inbred strains initially used in this work, Hd-rR and HNI, provide an excellent resource to study this problem as the genetic divergence between the two inbred strains is very high (SNP rate of ~3.42 %). Using another medaka inbred strain HSOK as an outgroup to Hd-rR and HNI (Fig. 3a), we were able to obtain inferences for the ancestral bases of Hd-rR and HNI at positions where multiple alignments of the three strains were available (Methods). We compared the ancestral base X with the current Hd-rR base Y at each position to calculate the mutation rate of base change from X to Y at each position within 500 bp from all representative TSSs. We observed that the sequence anisotropy around TSSs in each group was conserved from the common ancestor to Hd-rR, with net average mononucleotide increase/decrease incidences quite small (Fig. 3a) both overall around the TSS and as a function of position (Additional file 5: Figure S6b-d), although we saw a small increase in A/T incidences and a small decrease C/G incidences in testes, blastulae and liver (Fig. 3a, Additional file 6: Figure S9a and Additional file 7: Figure S10a). The base changes are more pronounced in downstream regions than in upstream regions.

Consistent with previous findings across all categories of TSS [9, 13], investigations of the mutagenic landscape confirm that single nucleotide mutation rates and insertion/deletion (indel) rates had chromatin-associated ~200-bp periodic patterns downstream of nucleocyclic TSSs in the testes, blastulae, and liver (Additional file 8: Figure S7). Higher genetic activity of nucleocyclic TSSs compared to other TSSs (Fig. 2c) might induce higher mutation and indel rates around nucleocyclic TSSs.

We then analyzed individual base change categories to determine how sequence variation during evolution might contribute to the conservation of the clear sequence composition boundaries around nucleocyclic TSSs. At each position in the genome, we calculated the ratio of the count of each base change X to Y to the “total” number of bases at the position. This normalization allows us to compare the rates of a mutation (e.g., C to T) and its opposite direction (e.g., T to C), indicating a drive toward the observed sequence features both upstream and downstream of nucleocyclic TSSs (Fig. 3b). The base composition drive is supported in each case of a reciprocal G/C to A/T pair, in that the corrected forward rate (G/C to A/T) is higher than the corrected reverse rate (A/T to G/C) downstream of nucleocyclic TSSs, yielding the small increase in A/T incidences and decrease in G/C incidences shown in Fig. 3a. We then compared the flow into each mononucleotide (e.g., the sum of average mutation rates from C, G, or T to A) and the flow out from the mononucleotide (e.g., from A to C, T, or G) in respective upstream and downstream regions. For this analysis, the normalization also facilitates the calculation of the flow into A, for example, as the sum of the breakdown of base change rates (C to A, G to A, and T to A) as illustrated in Fig. 3c. Figure 3c shows that, although rates of three mutations in the flows vary widely, we observe a bidirectional, although not completely balanced, flow into and out from each mononucleotide, contributing to the conservation of the mononucleotide incidence from the common ancestral genome to the Hd-rR genome (Fig. 2d). The small net flux toward A/T and away from G/C is notable in each case and would be expected to result in a net flux of genome sequence around nucleocyclic TSSs.

Figure 3d and Additional file 9: Figure S8a display a more precise picture of mutation rates at individual positions within 500 bp of nucleocyclic TSSs using data from testes. Figure 3d suggests that around the first nucleosome downstream of the TSSs, rates of C to T, and T to C are maximal around the nucleosome dyad and minimal around the linker, motivating us to verify this trend around first downstream nucleosomes (Fig. 3e). Remarkably, this tendency is significantly contrary to the general trend of genome-wide stable nucleosomes where rates of C to T and T to C base changes are minimal around the nucleosome dyad and maximal around the linker in the medaka genome (Fig. 3e, *p* = 0.02 % and 3.14 % for C to T and T to C, respectively by one-tailed Wilcoxon’s ranksum test) and in the human genome [14]. The trend is also seen using data from liver (Additional file 7: Figure S10e). Other mutational classes that have the tendency in testes, blastulae, and liver are A to T, T to A, and G to T (*p* < 5 %, Additional file 9: Figure S8, Additional file 6: Figure S9 and Additional file 7: Figure S10).

Another atypical mutation pattern seen with nucleocyclic TSSs was that rates of C to T base change were significantly higher than rates of G to A mutation downstream of nucleocyclic TSSs in testes and blastulae, which maintain germline character (Additional file 10: Figure S11a-b, *p* < 5 %, Methods). This pattern suggests a transcription-based polarity and a possible involvement of transcription-coupled DNA repair (TCR) that could skew mutational spectra and/or protects transcribed regions from mutations [38–41], resulting in an excess of C to T mutations over G to A mutations downstream of TSSs [40, 41]. One underlying feature that might lead to such a pattern would be an ability of TCR to suppress mutations particularly effectively downstream of nucleocyclic TSSs (Additional file 8: Figure S7).

## Conclusions

Substantial information on TSSs and nucleosome positioning from two highly divergent inbred medaka strains provided novel insights into a class of nucleocyclic TSSs with relevance to transcriptional activity during embryogenesis, lineage separation, and genetic variation. We revealed atypical evolution around nucleocyclic TSSs, which have higher gene expression and a clear boundary in sequence composition with potentially-nucleosome-destabilizing A/T-enrichment of upstream and nucleosome-accommodating C/G-enrichment downstream of the TSSs. We found that the sequence anisotropy is highly conserved from the common ancestor to Hd-rR due to near-equilibrium between the rates of specific mutation classes and their opposite counterparts. Downstream of nucleocyclic TSSs, the rates of C to T, and T to C increase around the first nucleosome dyad and decrease around the first linker, in contrast to base changes around genome-wide stable nucleosomes not selected for promoter presence, and C to T rates are higher than G to A rates, which suggests the involvement of transcription-coupled repair.

Several different processes might contribute to the associations between TSS expression and local discontinuity in sequence composition. We propose the following as possible ways that these processes may interact:

1. The initial position of some TSSs may be set by DNA-binding factors that serve as recruitment sites for RNA polymerase, with the initial pre-polymerase complex or the resulting polymerase-containing complex serving as an organizing boundary for subsequent nucleosome deposition.
2. Along with functional roles for sequences in recruiting and positioning polymerase, it remains possible that conservation of a TSS could arise solely as the result of barriers formed by discontinuities in DNA sequence composition, potentially as a result of DNA translocations that juxtapose sequences with very different characteristics.
3. However such a situation arises, an A/T nucleotide enrichment upstream of nucleocyclic TSSs should contribute to the eviction of nucleosomes, while a C/G-rich downstream of the TSSs may facilitate both occupancy and anchoring by arrays of positioned nucleosomes.
4. Any nucleosomal or sequence-composition discontinuity may feedback (in evolutionary time) toward favoring biased mutagenic “drift” in each domain that could retain the overall sequence organization. Such a passive stabilizing effect, combined with any active selection for maintenance of the TSS, would combine to produce long-term stability in the 5′ structure of specific genes.

## Methods

### Animal ethics statement

All experimental procedures and animal care were approved by the animal ethics committee of the University of Tokyo (approval number: 14–5).

### RNA-seq

We isolated RNA from single-cell-stage embryos, blastulae, and adult liver using the RNeasy mini kit (QIAGEN) or ISOGEN (NIPPON GENE) according to the manufacturer’s protocol. We treated purified RNAs with Ribominus eukaryote kit for RNA-seq (Life Technologies), and conducted RNA-seq analysis basically according to the manufacturer’s instructions. We performed sequencing on a HiSeq2000 platform (Illumina) using a TruSeq Cluster generation kit and SBS kit (version 3). We generated at least 20 million sequences of 36-bp per sample. After removing low-quality 36-bp reads with five or more undetectable bases, we mapped the remaining reads using the Burrows-Wheeler Aligner allowing no more than three mismatches and no gap, and used uniquely mapped reads for further analysis.

We investigated whether single-cell-stage embryos had characteristics similar to blastulae in terms of gene expression as embryonic stem cell lines can be established from blastulae [42]. Indeed, comparison among RNA-seq data from single-cell-stage embryos, blastulae, and liver showed that the Pearson’s correlation coefficient between RNA-seq data of single-cell-stage embryos and blastulae was 0.67, much higher than the correlation coefficient (0.05) between the single cell stage and liver. Assuming that blastulae maintain a zygotic character, we examined blastulae, testes, and liver to identify features around TSSs that are associated with consistently stable or tissue-specific expression.

### Collecting mRNA 5′-end tags and their reproducibility

We collected mRNA 36-nt 5′-end reads from three tissues (blastulae, testes, and liver) from Hd-rR and HNI medaka strains using an Illumina GAIIx sequencing platform according to the protocols described in ref. [9], which required 10 ug of total RNA. We used 3–5 million cells because one million cells yielded 2–4 ug total RNA. In terms of the reproducibility of the method for collecting 5′-end reads, we found a high correlation between technical replicates and validated the method using quantitative real-time PCR in our previous paper [32]. In addition, we performed two independent experiments on 2-day-old embryos (Additional file 11: Figure S2 in ref. [9]) and observed an extremely high correlation between the frequencies of individual 5′-end tags aligned to the genome (Pearson’s correlation coefficient = 0.996), which can be found in the section titled “Methods of collecting 5′-end tags and their reproducibility” in the supporting online material of ref. [9]. We validated the method using quantitative real-time PCR.

### TSS clusters and representative TSSs

The positions of the 25-nt 5′-end reads in the Hd-rR and HNI genomes were determined for reads that aligned to unique positions, allowing for a maximum of two mismatches, according to a previously described method [9]. We then defined TSS clusters and representative TSSs in the Hd-rR and HNI genomes, as described below:

1. For ease of comparison between tissues, the number of 5′-end reads aligned with each genomic position was normalized so that the total number of reads became 300,000, applying, for the purposes of display and discussion, the convention above that assigns a single cell transcriptome to ~300,000 mRNA molecules [33].
2. To obtain representative TSSs from the three samples (blastulae, testes, and liver), we merged the three tissue-specific expression levels from the three samples at each position by taking the average of the values. We then grouped proximal genomic positions with expression scores of > 0.1 that were within a distance of 20 bp into a TSS cluster. We used 0.1 as the cutoff value for the minimum normalized expression level because it was equivalent to two among 6 million 5′-end tags. Note that 5.5 - 9 million tags were collected from each of six tissues (see Table 1).
3. For the blastulae, testes, and liver, a representative TSS was defined as the position with the highest expression score among TSS clusters within 500 bp, and >500 bp apart from any neighboring representative TSSs to minimize neighboring TSS effects on nucleosome positioning.
4. In the three tissues, the TSS clusters and their representative TSSs defined above were used in common. A representative TSS could be either active or inactive in a focal tissue type, where a TSS was defined as active if its expression level was ≥ 0.1 in the tissue.

### Nucleosome positioning information

Mononucleosome cores were isolated from MNase-digested chromatin as previously described [4, 6]. We sequenced ends of cores using an Illumina HiSeq2500 sequencing platform to obtain 339–587 million 25-nt single-end reads for each tissue type, 66 % of which mapped to unique genomic positions, yielding 46-72-fold coverage of the entire genome for each tissue type (Table 2), which was sufficient to estimate nucleosome positioning. The degree of nucleosome dyad presence, which provided a local dyad positioning score, was measured from the positions where the nucleosome end reads were anchored, according to a method described previously [9]. We then smoothed the raw local dyad positioning score at each position by replacing it with a running average over a 21-bp window around the position.

### Estimating the average nucleosome core fragment length

We estimated the average nucleosome core fragment length as the distance between the ends of nucleosome core fragments that minimizes the discrepancy between the A/T mononucleotide distributions around nucleosome cores in the forward and reverse strands. The A/T distribution is only considered because it is complementary to the C/G distribution. Let ${R}^{+}$ be the set of reads that are anchored on the forward strand of the focal genome, while ${R}^{-}$ be those on the reverse strand. Let $L$ denote a candidate of average nucleosome core fragment length. For a read $r\in {R}^{+}$ that is aligned at position $x$, the nucleosome core fragment of $r$ starts from $x$ and ends at $x+L-1$. We then define the A/T distribution on the region that excludes 30 bases at both ends of a nucleosome core fragment to avoid the effect of the sequence composition skew, A/T followed by C/G, at MNase cleavage sites. Precisely, let ${\overrightarrow{v}}_{+,AT,L,r}$ be the vector of A/T nucleotide occurrences such that ${\overrightarrow{v}}_{+,AT,L,r}\left[i\right]$ is 1 if A or T is present at position $x+30+i$ for $i=0,\dots, L-61$, and is 0 otherwise. The range of $i$ is of size $L-60$. Reads associated with a nucleosome core do not always map to a consistent starting position but to multiple positions. The above definition allows that each read $r$ can have its own sequence distribution downstream of its starting position.

Let ${\overrightarrow{v}}_{+,AT,L}$ be the sum of vector ${\overrightarrow{v}}_{+,AT,L,r}$ for all $r\in {R}^{+}$ divided by the number of reads, ${\sum}_{r\in {R}^{+}}{\overrightarrow{v}}_{+,AT,L,r}/\left|{R}^{+}\right|$, which expresses the A/T mononucleotide “incidence” distribution around nucleosome cores in the forward strand. Let ${\overrightarrow{v}}_{-,AT,L,r}$ denote the A/T incidence distribution in the reverse strand. We define the discrepancy between the vectors in the forward and reverse strands as the mean squared distance; namely, ${\sum}_{i\in 0,\dots, L-61}{\left({\overrightarrow{v}}_{+,AT,L}\left[i\right]-{\overrightarrow{v}}_{-,AT,L}\left[i\right]\right)}^2/\left(L-60\right)$. Recall that $i$ ranges from 0 to $L-61$ because the range size in the denominator is $L-60$. We then select the optimal length $L$ that minimizes the discrepancy between ${\overrightarrow{v}}_{+,AT,L}$ and ${\overrightarrow{v}}_{-,AT,L}$ within the range of $147 - 10\le L\le 147+10$, where 147 and 10 approximate the length of DNA wrapping around one nucleosome core and the length of one helical turn. Additional file 11: Figure S2d-i show that the optimal lengths in testes, blastulae, and liver are 148, 150, and 148, respectively, and in blastulae, 148 is the second best. Because these optimal lengths were close to 147, the previously known size of one nucleosome core, we used 147 bp as the value of nucleosome core length in our analysis.

### Selecting stable nucleosome dyads in the entire genome

In each tissue type (testes, blastulae, and liver), we selected “stable” nucleosome dyads that had proximal dyads in the other two tissue types, according to the following procedure:

1. We calculated local dyad positioning scores in the forward and reverse strands, separately, and used the scores in the forward strand.
2. In each tissue type, we selected candidate nucleosome dyads that maximized local dyad positioning scores within $L$ bp of their positions, where we set the value of $L$ to 147 based the analysis of optimal values of average nucleosome core fragment length. Afterwards, a peak was retained if it had no other peaks within 165 bp of its position, where 165 bp is the average distance between neighboring nucleosome dyads in the medaka genome [9].
3. From the candidate nucleosome dyad positions, we eliminated such dyads that their surrounding regions within 165-bp of their positions were not unique and had a repetitive 25-bp region that mapped to another position in the genome. This step is effective in removing false-positive dyads.
4. From the remaining candidate nucleosome dyads in each tissue type, we selected those dyads that had proximal dyads within 10 bp distance from them in the other two tissue types. The selected dyads were defined as the stable nucleosome dyads in the focal tissue type.

The respective numbers of stable nucleosome dyads in testes, blastulae, and liver were 48,887, 45,499, and 45,312. Around these stable nucleosome dyads, we examined mononucleotide incidences (Fig. 2f, Additional file 2: Figure S4d, and Additional file 3: Figure S5d) and mutation rates (Fig. 3e, Additional file 9: Figure S8b, Additional file 6: Figure S9e, and Additional file 7: Figure S10e).

### Locating first nucleosome dyads downstream of TSSs

Since Fig. 2a, Additional file 2: Figure S4a, and Additional file 3: Figure S5a show that the first nucleosome dyads downstream of representative TSSs are located at ~100 bp downstream on average, for each representative TSS, we defined the first nucleosome dyad as the position with the maximal local dyad positioning score in the region [+50, +150] downstream of the representative TSSs (see Fig. 2e). The first dyad was undefined if all scores in the region were zero, though the number of such cases was quite small (<0.25 %). When more than one TSS share the same first nucleosome dyad, only one occurrence of the dyad was considered.

### Measuring the periodicity and consistency of arrays of nucleosomes upstream or downstream of a representative TSS using autocorrelation

Let $s(x)\;\left(0\le s(x)\le 1\right)$ denote the smoothed local dyad positioning score of a nucleosome at position $x=-500,\dots, +500$ within 500 bp from a representative TSS, where $x=0$ is the position of the representative TSS. Intuitively, the autocorrelation, denoted $R(L)$, is the sum of similarities between $s(x)$ and $s\left(x-L\right)$ as a function of lag $L$ that is supposed to represent the periodicity of nucleosomes (see a formal definition of autocorrelation in [43]). We calculated $R(L)$ using the acf function available in R. Autocorrelation $R(L)$ changes according to the value of $L$ and is expected to be maximal for the inherent periodicity of nucleosomes. Thus, when $R(L)$ is maximal for *L* = 150~220, we define $L$ as the periodicity and $R(L)$ as the consistency of arrays of nucleosomes upstream (or downstream) of a representative TSS. We searched the range 150~220 because we found that most of optimal periodicities ranged from 160 to 210 bp from preliminary experiments.

### One-to-one reciprocally best pairs of alignments between the Hd-rR and HNI genomes

We performed a *de novo* assembly of the HNI genome using 453,278,992 paired-end 76-nt Illumina reads, which amounted to ~43-fold coverage of the entire HNI genome when the genome size was assumed to be 800 Mb. Specifically, we used ABySS [44] and set the *k*-mer length at 50. The N50 length was 2707 bp, and the maximum contig size was 61,228 bp. The HNI contigs were aligned to the Hd-rR scaffolds using BWA-SW [45] with default parameters. Reciprocally best pairs of alignments were retained for downstream analyses. According to the alignments, the representative TSSs in the Hd-rR genome were mapped to the HNI genome coordinate. The representative Hd-rR TSSs mapped to the HNI genome were associated with their most proximal representative TSSs in the HNI genome, and the distance between each pair was calculated. Similarly, the representative HNI TSSs were mapped to the Hd-rR genome and were associated with the distances to the nearest representative Hd-rR TSSs.

### Determining the ancestral bases of Hd-rR and HNI using HSOK as an outgroup

We collected 132,389,894 76-nt Illumina reads from another medaka inbred strain HSOK, an outgroup to Hd-rR and HNI (Additional file 1: Figure S1a), to determine 448,535,031 ancestral bases of Hd-rR and HNI at positions where multiple alignments of the three strains were available.

### Parameters associated with a representative TSS

We associated the following parameters with 12,347 representative TSSs in Fig. 1b. Some parameters have missing values because the draft genomes of Hd-rR and HNI are partial and have many gaps. We therefore eliminated TSSs with missing values to obtain a set of 11,336 TSSs with complete information, and calculated Spearman’s rank correlation coefficient between pairs of the parameters for the dataset, as shown in Fig. 1d.

- Expression level of a representative TSS in each of the three tissue types: This is the normalized sum of 5′-end tags in the TSS cluster for a representative TSS assuming that the total number of tags in one cell is 300,000. The value is undefined when no 5′-end tags are found in one of the tissues.
- Breadth of the TSS cluster for a representative TSS in each of the three tissue types: To eliminate noise at both ends of each TSS cluster, we calculated the cumulative sum of 5′-end tags from 5′-end to 3′-end, used the positions associated with the 5th and 95th percentiles of the cumulative sums as the 5′-end and 3′-end of the cluster, respectively. The distance between the ends is defined as the breadth. After this treatment, remarkably, the breadth values in the three tissue types coincide in most cases, except that the distance is zero when the gene for the cluster is not expressed in a particular tissue.
- Periodicity and consistency of nucleosome positioning in the two regions upstream [−500,0] and downstream [0,+500] of a representative TSS: The periodicity of nucleosome positioning surrounding each TSS changes. Even in one TSS, the periodicities upstream and downstream of the TSS can differ greatly. We examined the two separate regions within 500 bp upstream and downstream of each TSS, and calculated the optimal periodicity associated with the maximal auto-correlation value in each region. We computed these values for blastulae, testes, and liver in the Hd-rR and HNI strains.
- Sequence asymmetry values of dinucleotide counts surrounding a representative TSS: The respective numbers of dinucleotide content (AA or TT denoted by AA_TT, CC or GG by CC_GG) and single nucleotide content (A, C, G, T, A or T represented by A_T, C or G by C_T) were counted 500 bp upstream and downstream of each representative TSS. The sequence asymmetry values are defined as the simple differences between upstream and downstream counts. A negative value indicates that the dinucleotide count is higher in the downstream region than in the upstream region.

### Comparison of parameters between nucleocyclic and non-nucleocyclic TSSs

We examined whether the parameter distributions of the nucleocyclic TSSs in Group 1 were larger (or, smaller) than those of the non-nucleocyclic TSSs in Groups 2 and 3 using one-tailed Wilcoxon’s ranksum test of the null-hypothesis that the parameter distributions in the two populations are equal. We used Wilcoxon’s ranksum test, a non-parametric test, for this analysis because the distribution of each parameter is not necessarily normal. We applied this test after preprocessing the parameters: gene expression, and distance between pairs of representative TSSs.

- For gene expression and TSS breadth, we tested whether the gene expression and TSS breadth distributions of the nucleocyclic TSSs were larger than those of the non-nucleocyclic TSSs. Figure 2c, Additional file 2: Figure S4b and Additional file 3: Figure S5b show the analysis of gene expression.
- To examine if nucleocyclic TSSs are more positionally conserved between Hd-rR and HNI than non-nucleocyclic TSSs, for distance between pairs of representative TSSs in the two strains, we tested the difference between the distance distributions of the nucleocyclic TSSs and non-nucleocyclic TSSs in each of the three tissue types, separately.

### Significance of asymmetry of base incidence around nucleocyclic TSSs

We measured the significance of asymmetry of the focal base between 500 positions upstream and downstream of nucleocyclic (or, non-nucleocyclic) TSSs using a two sample *z*-test of the null-hypothesis that the average base incidences upstream and downstream are equal. A lower *p*-value indicates a higher asymmetry. Precisely, we counted the focal base frequency (denoted by ${n}_1$ and ${n}_2$) upstream and downstream of the TSSs and the total numbers of four bases (${m}_1$ and ${m}_2$) upstream and downstream of the TSSs. We then defined the average nucleotide incidence as $\mu =\left({n}_1+{n}_2\right)/\left({m}_1+{m}_2\right)$, and the *z*-value as $Z=\frac{n_1/{m}_1-{n}_2/{m}_2}{\sqrt{\mu \Big(1-\mu \Big)\left(\frac{1}{n_1}+\frac{1}{n_2}\right)}}$. Since *z*-values follow the standard normal distribution, their *p*-values can be calculated accordingly.

### Computing mononucleotide (or, mutation) incidences around the first nucleosome dyads downstream of TSSs and those around nucleosome dyads in the entire genome, considering MNase sequence preferences

In the case of the first nucleosome dyads downstream of TSSs, we only considered the transcribed strand downstream of TSSs and calculated mononucleotide and mutation rates. In contrast, around nucleosome dyads in the entire genome, we considered both of the plus and minus strands and averaged the mononucleotide and mutation rates in both strands so that the incidences of complementary bases (such as A and T) and complementary mutations (such as A->C and T->G) mirror horizontally around dyads. In this calculation, to minimize the effect of sequence preferences by MNase that cleaves DNA at A or T nucleotides, we examined how the base and mutation rates changed around the first linker upstream of dyads and found that the rates at 70–74 bp upstream of dyads were substantially different from their neighboring rates. We therefore excluded the range (70–74 bp upstream of dyads) from consideration, but we complemented and displayed the mononucleotide and mutation rates in the range in Fig. 2f, Additional file 2: Figure S4d and Additional file 3: Figure S5d because we smoothed the rates using a running average over 41-bp window.

### Significance of the differences of mononucleotide (or, mutation) incidences around the first nucleosome dyads and linkers downstream of nucleocyclic TSSs and those around nucleosome dyads and linkers in the entire genome

We calculated the difference of average mononucleotide (or, mutation) incidences at each position around first nucleotide dyads downstream of nucleocyclic TSSs and around dyads in the entire genome to generate the difference distribution around the two types of nucleosome dyads. We then used one-tailed Wilcoxon’s ranksum test to examine the null hypothesis that the difference distribution within 30 bp around nucleosome dyads was equal to the difference distribution within 30 bp around putative nucleosome linkers 100 bp downstream of dyads because nucleosome linkers are located at 100 bp downstream positions on average. The distance of 30 bp was used to separate the dyad and linker regions with a large margin of 40 bp in size while retaining a sufficient number of data at 61 positions for statistical analysis. Figure 2f, Additional file 2: Figure S4d, and Additional file 3: Figure S5d show the significance of the “mononucleotide” incidence difference. Figure 3e, Additional file 9: Figure S8b, Additional file 6: Figure S9e and Additional file 7: Figure S10e present the significance of the “mutation” rate difference.

### Comparison of C to T and G to A mutation rates between the 500 bp regions upstream and downstream of nucleocyclic TSSs

To examine the effect of transcription-coupled repair, we measured the difference of average mutation rates, C to T and G to A, at each position within 500 bp around nucleocyclic TSSs in the three tissue types, and tested whether the difference distribution upstream of nucleocyclic TSSs was smaller than the distribution downstream using one-tailed Wilcoxon’s ranksum test (see Additional file 10: Figure S11).

### Data availability

All sequence data are deposited at NCBI Archive (Study Accession SRP008998).

## Additional files

Additional file 1: Figure S1. a. Phylogenetic tree of HNI, Hd-rR, and HSOK, deriving respectively from northern Japanese, southern Japanese, and Korean medaka populations. b. Frequency distribution of distances between pairs of proximal representative TSSs in the Hd-rR and HNI genomes. c. Spearman’s rank correlation coefficient matrix for dinucleotide content (AA or TT denoted by AA_TT, CC or GG by CC_GG), single nucleotide content (A, C, G, T, A or T represented by A_T, C or G by C_G), and their asymmetry values (Sa) upstream and downstream of TSSs in the Hd-rR (Hd) and HNI (HN) strains. Many pairs of parameters are highly correlated positively (colored red) or negatively (green), implying considerable redundancy of parameters. We noticed that each parameter was highly correlated with one of the parameters in the yellow boxes (Hd/HN_A/C/G/T_Up/Dw, Hd/HN_Sa_A/C/G/T). To reduce the number of parameters, we selected to use the parameters in the yellow boxes for further analysis. d. Nucleosome coverage distribution of each tissue. The vertical axis displays the cumulative fraction of nucleotides covered by > x nucleosome cores where x is the coverage shown in the horizontal axis. (PDF 752 kb) Additional file 2: Figure S4. Characteristics of nucleocyclic TSSs in the blastulae. a. Representative TSSs in blastulae are clustered into three groups according to nucleosome positioning consistency (autocorrelation) downstream of the TSSs. The upper left box shows the number of TSSs in each group. A running average over 21-bp window is shown. b. Distribution of gene expression (TSS capture frequencies) in log scale. Capture frequencies for the nucleocyclic TSSs in Group 1 is significantly higher than that of non-nucleocyclic TSSs in Group 2 or 3 (*p* = 1.9 x 10^-5^). c. Average incidence of nucleobase A, T, C and G at positions within 500 bp from representative TSSs in Groups 1, 2 and 3. A running average over 41-bp window is shown. The small histogram in each graph shows the significance of asymmetry of each rate in individual group. The arrows suggest that the A, C, and G incidences around the first nucleosome dyads and linkers downstream of nucleocyclic TSSs significantly differ from those incidences around nucleosomes in the entire genome in Figure S4d. d. Around nucleosome dyads in the entire genome, the A/T (C/G, respectively) incidences around linkers are smaller (greater) than those around dyads, while this tendency is less pronounced around first dyads downstream of TSSs. Indeed, the difference in the tendency is significant; namely, *p* < 10^-6^ for any of A, C, G, and T by one-tailed Wilcoxon’s ranksum test (Materials and Methods). A running average over 41-bp window is shown. (PDF 821 kb) Additional file 3: Figure S5. Characteristics of nucleocyclic TSSs in the liver. a-d. The definitions are similar to those in Figure S4. In Figure b, expression of the nucleocyclic TSSs in Group 1 is significantly higher than that of non-nucleocyclic TSSs in Group 2 or 3 (*p*-value < 10^-6^). In Figure d, the difference in the tendency is significant; namely, *p* < 10^-12^ for any of A, C, G, and T. (PDF 822 kb) Additional file 4: Figure S3. Schematic illustration of asymmetric base composition coinciding with arrays of positioned nucleosomes downstream of nucleocyclic TSSs. (PDF 346 kb) Additional file 5: Figure S6. Average mononucleotide substitution rates from the common ancestor of Hd-rR and HNI to the current Hd-rR genome around nucleocyclic TSSs. a. Phylogenetic tree of HNI, Hd-rR, and HSOK. b-d. Average substitution rates at individual positions within 500 bp from nucleocyclic TSSs in testes (b), blastulae (c), and liver (d). A running average over 41-bp window is shown. (PDF 1325 kb) Additional file 6: Figure S9. Atypical evolution around nucleocyclic TSSs in blastulae. The definitions of graphs are similar to those in Fig. 3. (PDF 835 kb) Additional file 7: Figure S10. Atypical evolution around nucleocyclic TSSs in liver. The definitions of graphs are similar to those in Fig. 3. (PDF 829 kb) Additional file 8: Figure S7. Single nucleotide mutation rates and indel rates at positions within 500 bp around nucleocyclic/non-nucleocyclic TSSs. a-f. Single nucleotide mutation rates at positions within 500 bp of TSSs in testes (a), blastulae (c) and liver (e), and indel rates at positions within 500 bp of TSSs in testes (b), blastulae (d) and liver (f). A running average over 41-bp window is shown. (PDF 706 kb) Additional file 9: Figure S8. Atypical evolution around nucleocyclic TSSs in testes. a-b. The definitions of graphs are similar to those in Fig. 3d-e. (PDF 745 kb) Additional file 10: Figure S11. Possible effect of transcription-coupled repair (TCR). a-c. We observed a significant excess of C to T mutations over G to A mutations in transcribed regions downstream of nucleocyclic TSSs in testes (*p* = 3.70 % by one-tailed Wilcoxon’s ranksum test described in Methods, a) and in blastulae (*p* = 4.69 %, b). The *p*-value for the liver case is 37.92 %. (PDF 629 kb) Additional file 11: Figure S2. Estimating the average nucleosome core fragment length L. a. We calculated A/T mononucleotide distributions downstream of the start sites of nucleosome core fragment reads in the forward and reverse strands, separately. b. The A/T distributions of the forward and reverse strands in testes. c-e. We selected the value of L based on a best fit between the independent leading and lagging A/T base composition curves (b) on a genomewide scale, with a concordance in each case between a qualitative matching of the curves (d) and a quantitative “least squares” fit (a minimum discrepancy) between the two (e). Figure d shows the A/T distributions when L is set to the best fit (the minimum discrepancy), 148 bp, as shown in Figure e. f,g. The A/T distributions (f) for the best fit length 150 bp (g) in blastulae. h,i. The A/T distributions (h) for the best fit length 150 bp (i) in liver. (PDF 520 kb) Additional file 12: Table S1. (XLSX 81 kb)

## Abbreviations

TSS: Transcription start site
SNP: Single nucleotide polymorphism
W: A/T
S: C/G
