# Supplementary material for: Associations between nucleosome phasing, sequence asymmetry, and tissue-specific expression in a set of inbred Medaka species
Source: BMC Genomics. 2015 Nov 19;16:978. doi: 10.1186/s12864-015-2198-5 (PMC4653950; doi:10.1186/s12864-015-2198-5)
Supplement: Additional file 1: Figure S1. — a. Phylogenetic tree of HNI, Hd-rR, and HSOK, deriving respectively from northern Japanese, southern Japanese, and Korean medaka populations. b. Frequency distribution of distances between pairs of proximal representative TSSs in the Hd-rR and HNI genomes. c. Spearman’s rank correlation coefficient matrix for dinucleotide content (AA or TT denoted by AA_TT, CC or GG by CC_GG), single nucleotide content (A, C, G, T, A or T represented by A_T, C or G by C_G), and their asymmetry values (Sa) upstream and downstream of TSSs in the Hd-rR (Hd) and HNI (HN) strains. Many pairs of parameters are highly correlated positively (colored red) or negatively (green), implying considerable redundancy of parameters. We noticed that each parameter was highly correlated with one of the parameters in the yellow boxes (Hd/HN_A/C/G/T_Up/Dw, Hd/HN_Sa_A/C/G/T). To reduce the number of parameters, we selected to use the parameters in the yellow boxes for further analysis. d. Nucleosome coverage distribution of each tissue. The vertical axis displays the cumulative fraction of nucleotides covered by > x nucleosome cores where x is the coverage shown in the horizontal axis. (PDF 752 kb) [file 12864_2015_2198_MOESM1_ESM.pdf]

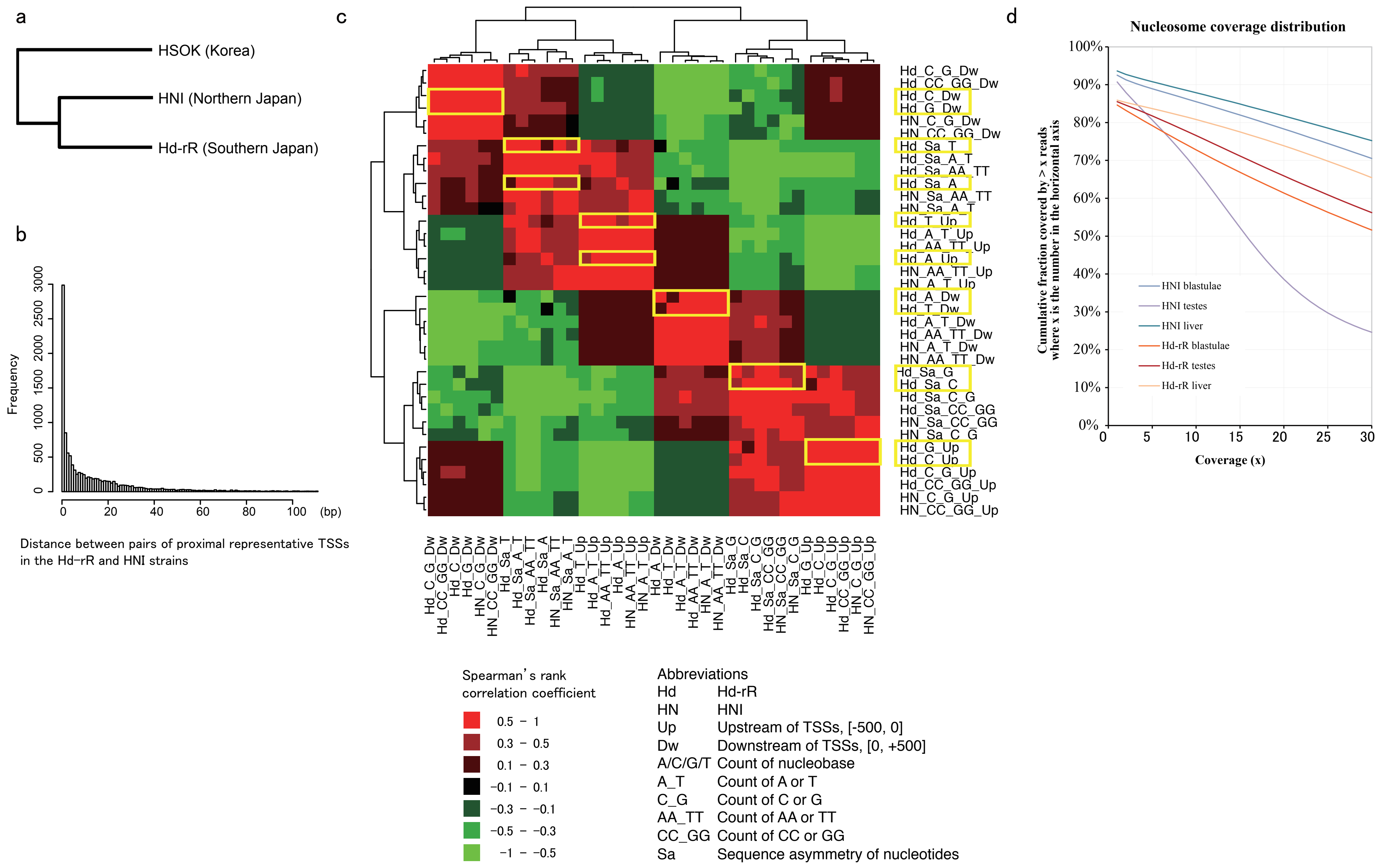
a
HSOK (Korea)
HNI (Northern Japan)
Hd-rR (Southern Japan)
b
Frequency
0
500
1000
1500
2000
2500
3000
0
20
40
60
80
100
(bp)
Distance between pairs of proximal representative TSSs in the Hd-rR and HNI strains
c
Hd_C_G_Dw
Hd_CC_GG_Dw
Hd_C_Dw
Hd_G_Dw
HN_C_G_Dw
HN_CC_GG_Dw
Hd_Sa_T
Hd_Sa_A_T
Hd_Sa_AA_TT
Hd_Sa_A
HN_Sa_AA_TT
HN_Sa_A_T
Hd_T_Up
Hd_A_T_Up
Hd_AA_TT_Up
Hd_A_Up
HN_AA_TT_Up
HN_A_T_Up
Hd_A_Dw
Hd_T_Dw
Hd_A_T_Dw
Hd_AA_TT_Dw
HN_A_T_Dw
HN_AA_TT_Dw
Hd_Sa_G
Hd_Sa_C
Hd_Sa_C_G
Hd_Sa_CC_GG
HN_Sa_CC_GG
HN_Sa_C_G
Hd_G_Up
Hd_C_Up
Hd_C_G_Up
Hd_CC_GG_Up
HN_C_G_Up
HN_CC_GG_Up
Spearman's rank correlation coefficient
0.5 – 1
0.3 – 0.5
0.1 – 0.3
−0.1 – 0.1
−0.3 – −0.1
−0.5 – −0.3
−1 – −0.5
Abbreviations
Hd Hd-rR
HN HNI
Up Upstream of TSSs, [-500, 0]
Dw Downstream of TSSs, [0, +500]
A/C/G/T Count of nucleobase
A_T Count of A or T
C_G Count of C or G
AA_TT Count of AA or TT
CC_GG Count of CC or GG
Sa Sequence asymmetry of nucleotides
d
Nucleosome coverage distribution
Cumulative fraction covered by > x reads where x is the number in the horizontal axis
100%
90%
80%
70%
60%
50%
40%
30%
20%
10%
0%
0
5
10
15
20
25
30
Coverage (x)
HNI blastulae
HNI testes
HNI liver
Hd-rR blastulae
Hd-rR testes
Hd-rR liver
