# Supplementary figures and images for: Associations between nucleosome phasing, sequence asymmetry, and tissue-specific expression in a set of inbred Medaka species
Source: BMC Genomics. 2015 Nov 19;16:978. doi: 10.1186/s12864-015-2198-5 (PMC4653950; doi:10.1186/s12864-015-2198-5)

a

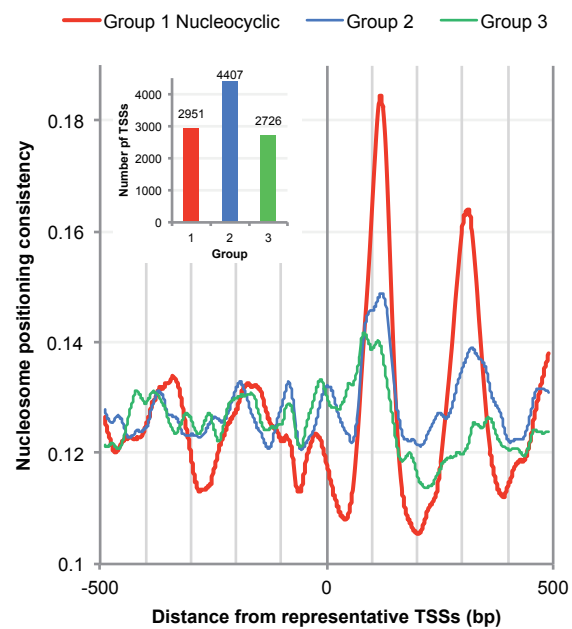

b

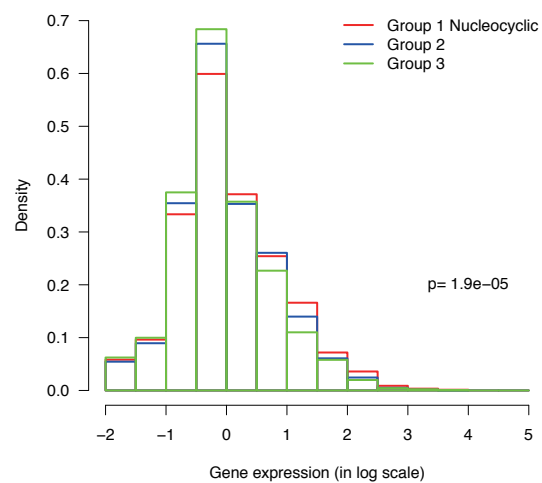

c

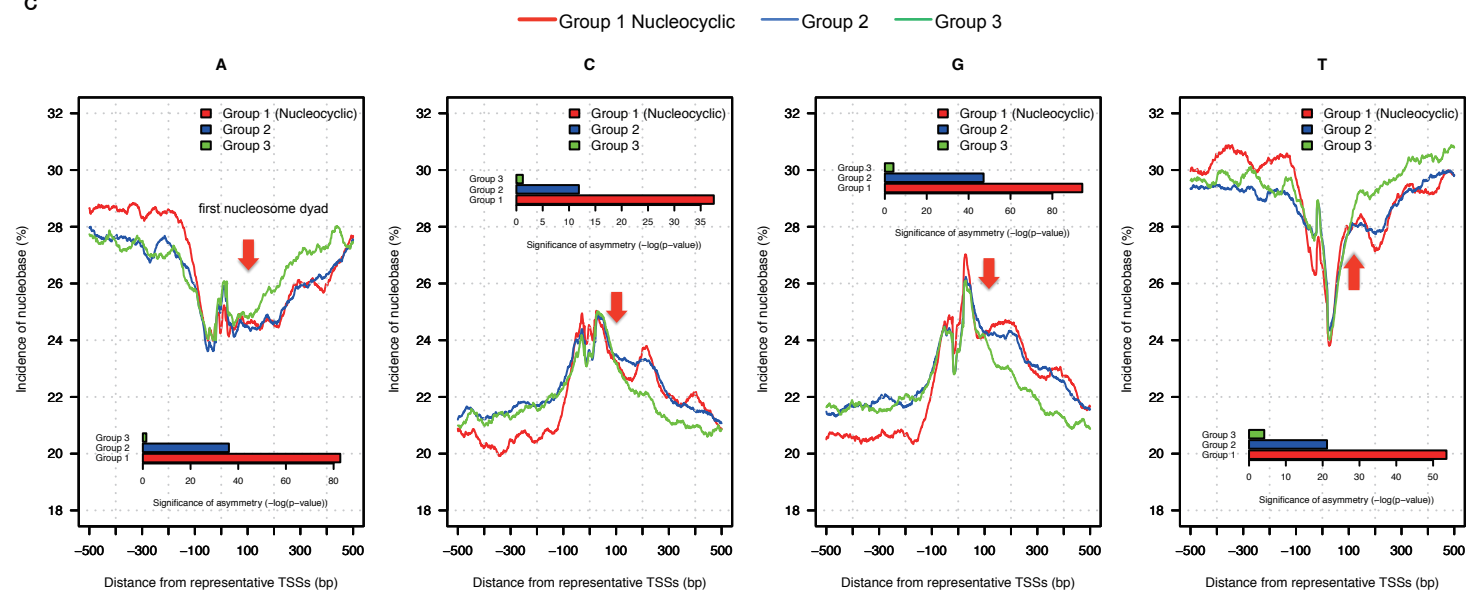

d

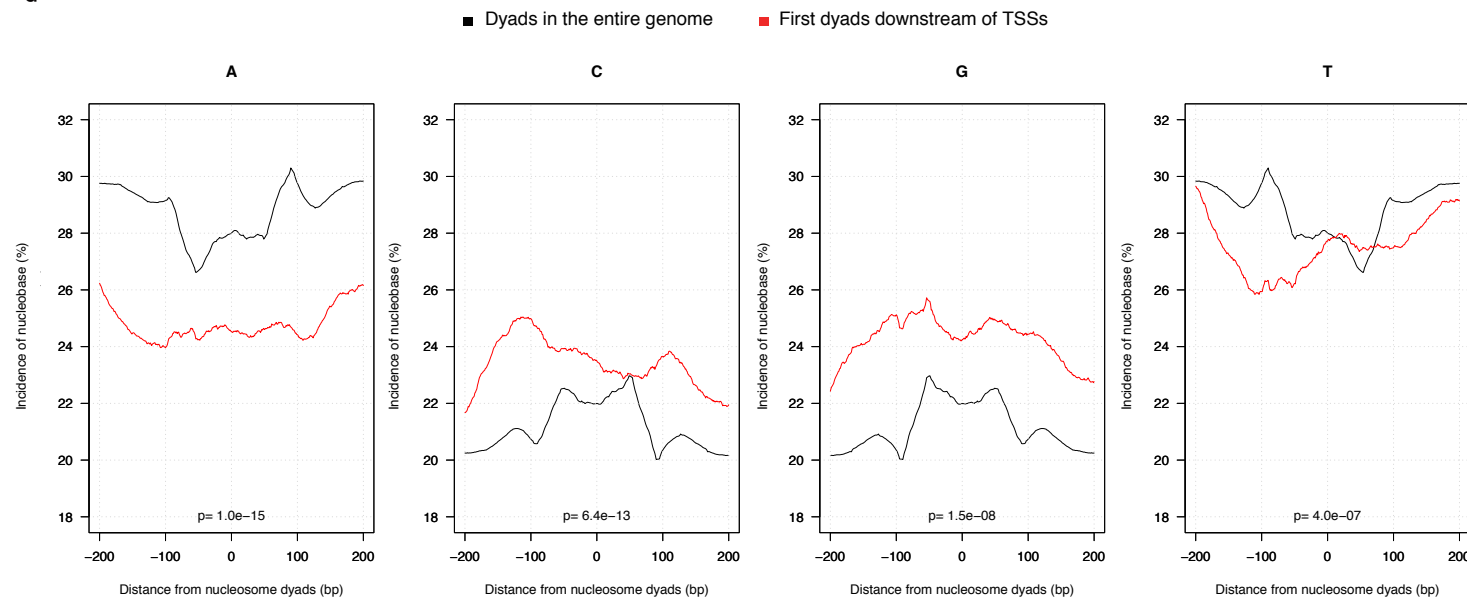

Supplement: Additional file 2: Figure S4. — Characteristics of nucleocyclic TSSs in the blastulae. a. Representative TSSs in blastulae are clustered into three groups according to nucleosome positioning consistency (autocorrelation) downstream of the TSSs. The upper left box shows the number of TSSs in each group. A running average over 21-bp window is shown. b. Distribution of gene expression (TSS capture frequencies) in log scale. Capture frequencies for the nucleocyclic TSSs in Group 1 is significantly higher than that of non-nucleocyclic TSSs in Group 2 or 3 (p = 1.9 x 10^-5^). c. Average incidence of nucleobase A, T, C and G at positions within 500 bp from representative TSSs in Groups 1, 2 and 3. A running average over 41-bp window is shown. The small histogram in each graph shows the significance of asymmetry of each rate in individual group. The arrows suggest that the A, C, and G incidences around the first nucleosome dyads and linkers downstream of nucleocyclic TSSs significantly differ from those incidences around nucleosomes in the entire genome in Figure S4d. d. Around nucleosome dyads in the entire genome, the A/T (C/G, respectively) incidences around linkers are smaller (greater) than those around dyads, while this tendency is less pronounced around first dyads downstream of TSSs. Indeed, the difference in the tendency is significant; namely, p < 10-6 for any of A, C, G, and T by one-tailed Wilcoxon’s ranksum test (Materials and Methods). A running average over 41-bp window is shown. (PDF 821 kb) [file 12864_2015_2198_MOESM2_ESM.pdf]

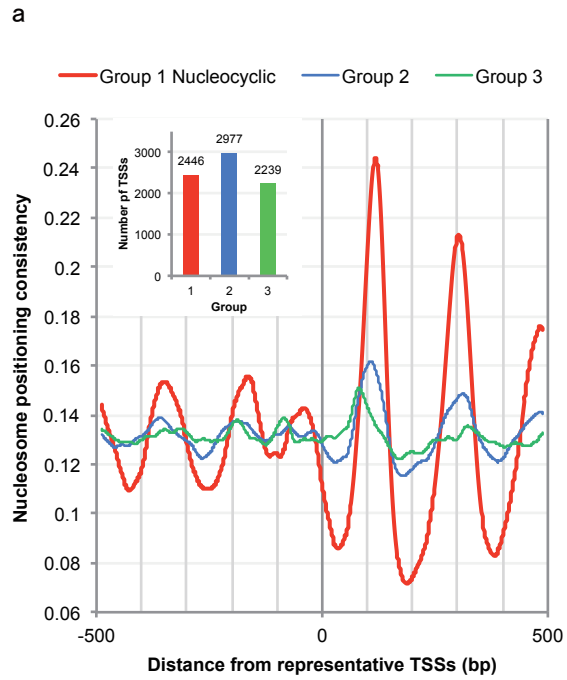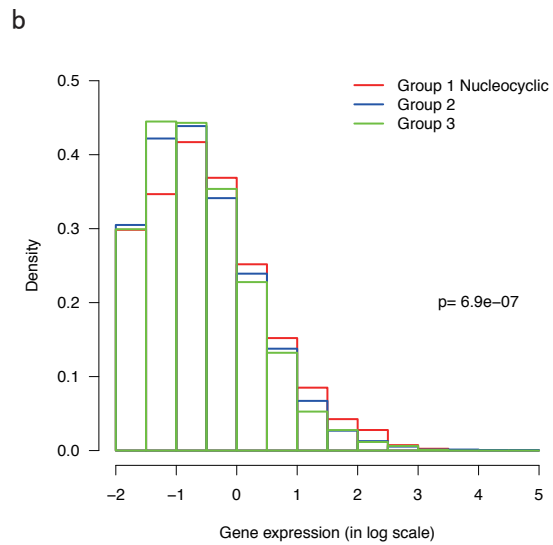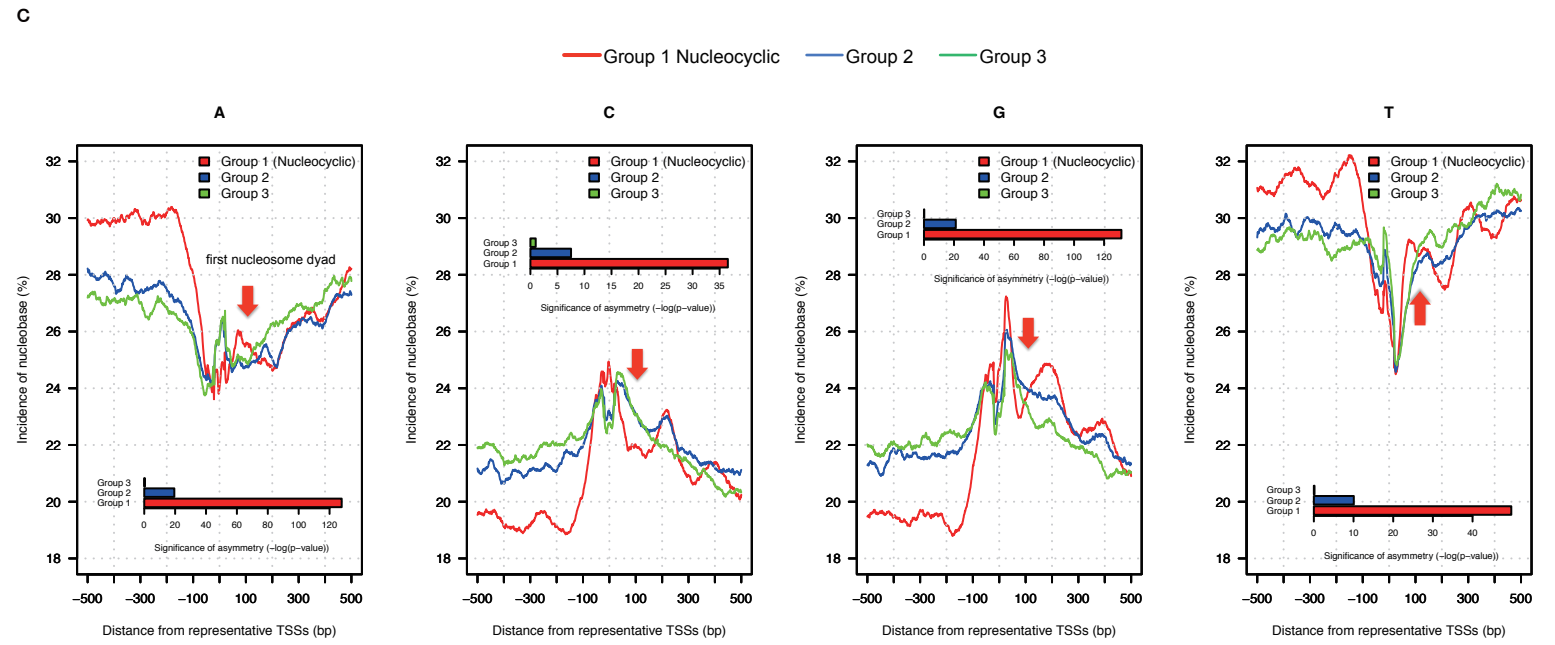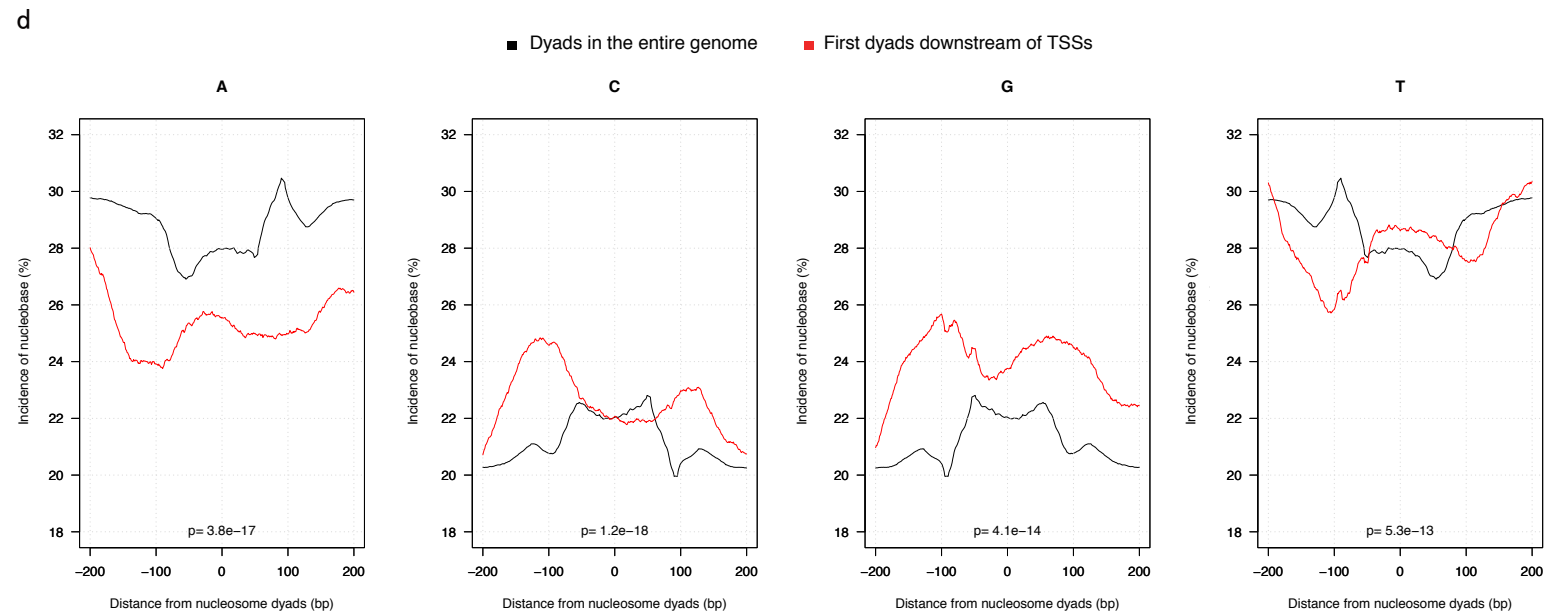

Supplement: Additional file 3: Figure S5. — Characteristics of nucleocyclic TSSs in the liver. a-d. The definitions are similar to those in Figure S4. In Figure b, expression of the nucleocyclic TSSs in Group 1 is significantly higher than that of non-nucleocyclic TSSs in Group 2 or 3 (p-value < 10-6). In Figure d, the difference in the tendency is significant; namely, p < 10-12 for any of A, C, G, and T. (PDF 822 kb) [file 12864_2015_2198_MOESM3_ESM.pdf]

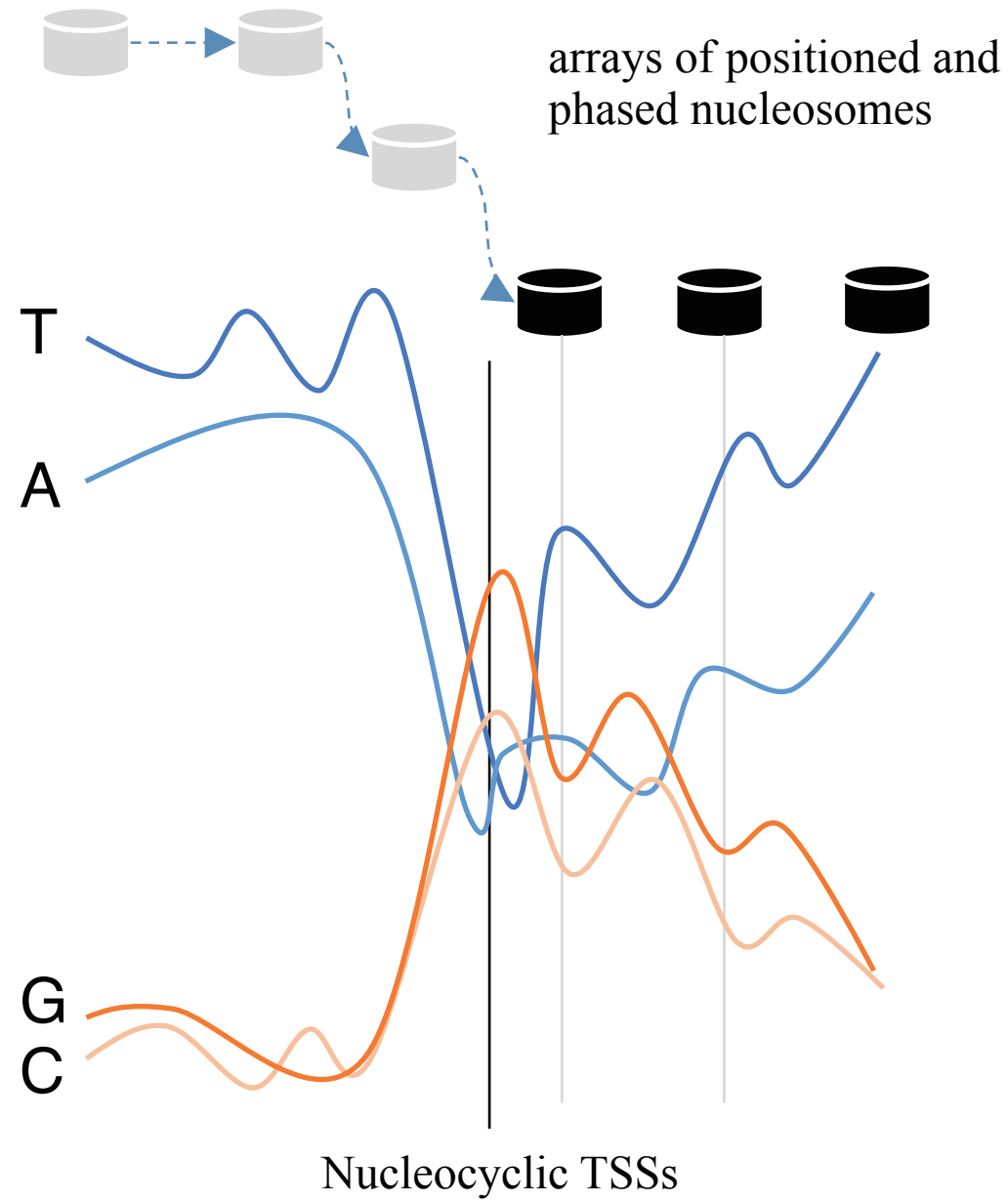

Supplement: Additional file 4: Figure S3. — Schematic illustration of asymmetric base composition coinciding with arrays of positioned nucleosomes downstream of nucleocyclic TSSs. (PDF 346 kb) [file 12864_2015_2198_MOESM4_ESM.pdf]

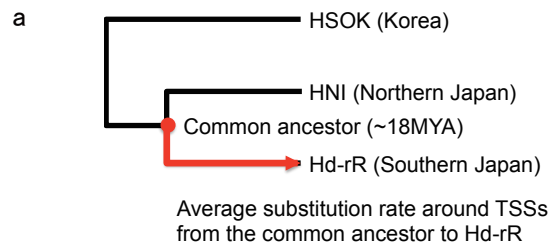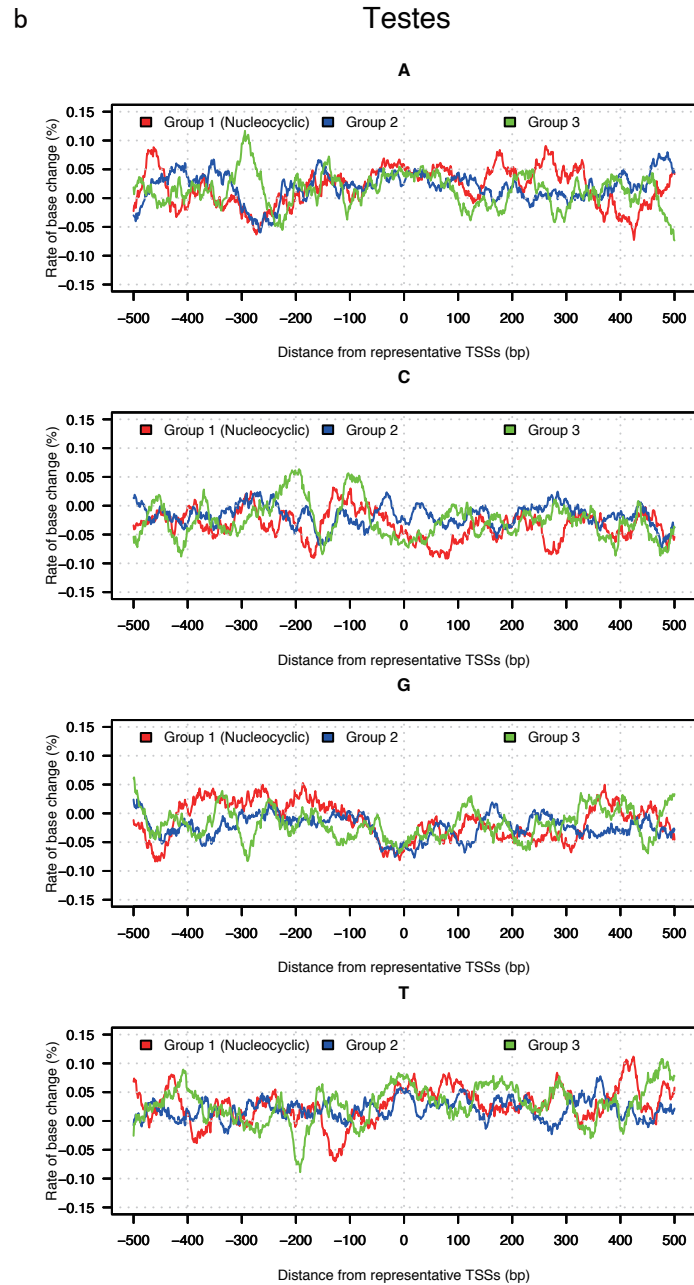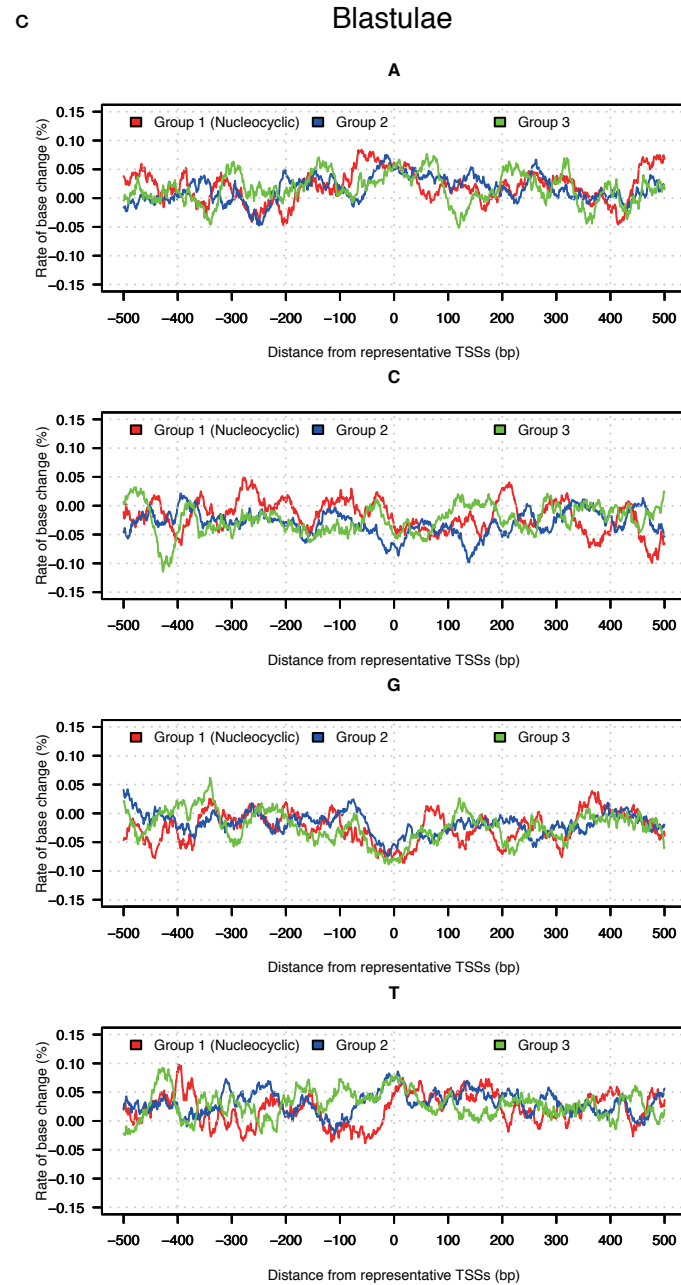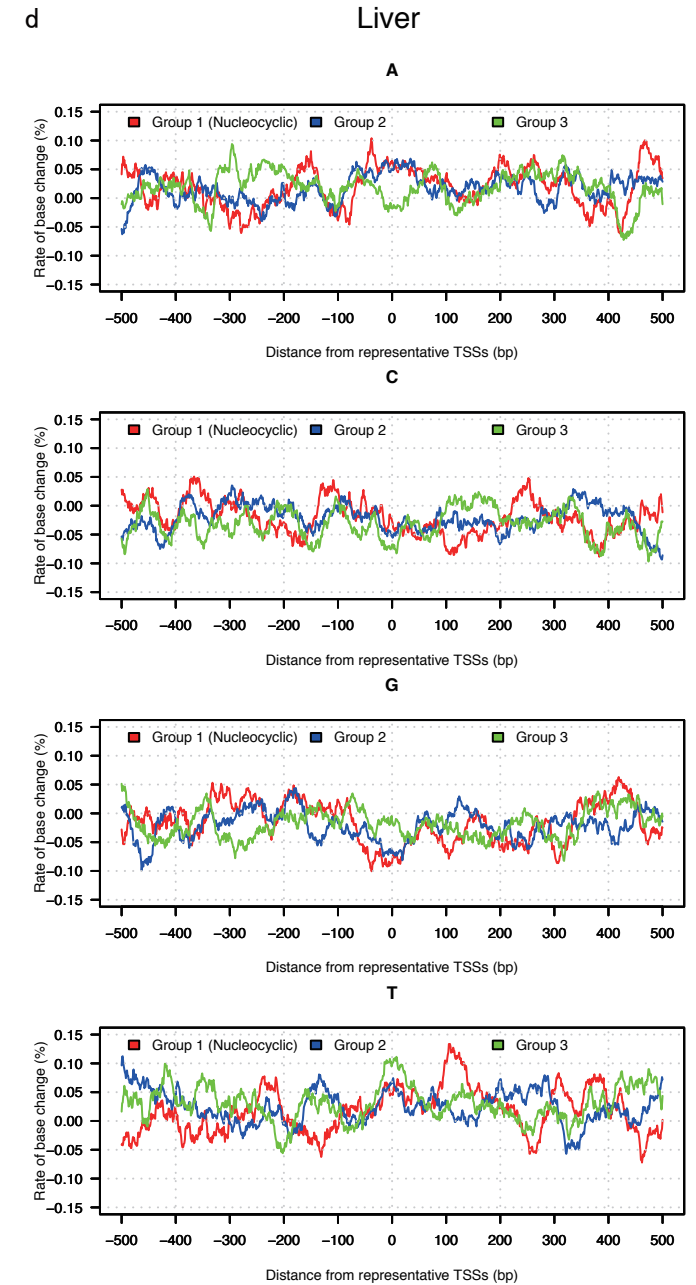

Supplement: Additional file 5: Figure S6. — Average mononucleotide substitution rates from the common ancestor of Hd-rR and HNI to the current Hd-rR genome around nucleocyclic TSSs. a. Phylogenetic tree of HNI, Hd-rR, and HSOK. b-d. Average substitution rates at individual positions within 500 bp from nucleocyclic TSSs in testes (b), blastulae (c), and liver (d). A running average over 41-bp window is shown. (PDF 1325 kb) [file 12864_2015_2198_MOESM5_ESM.pdf]

a

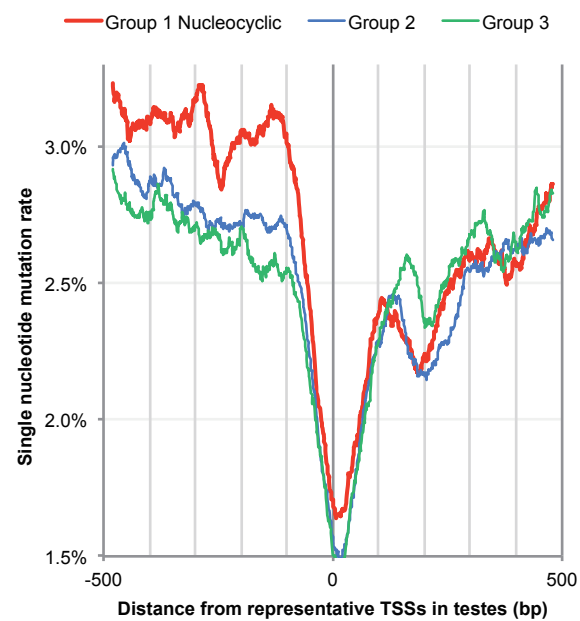

c

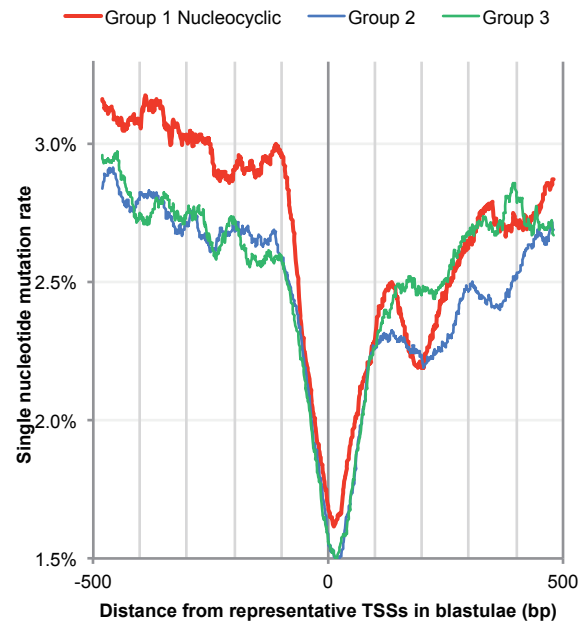

e

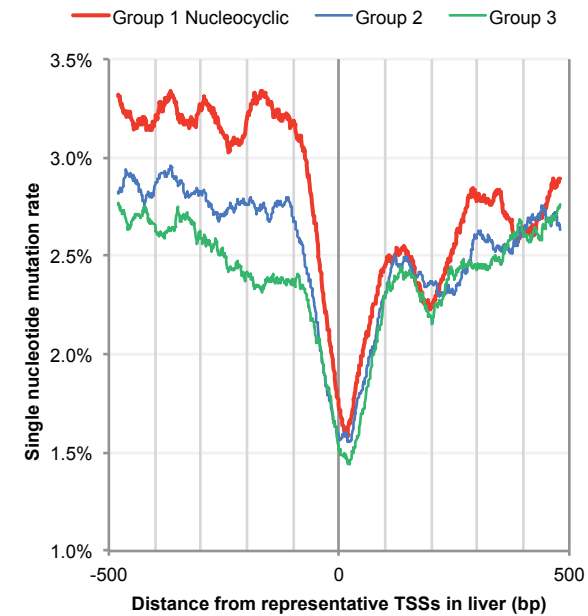

b

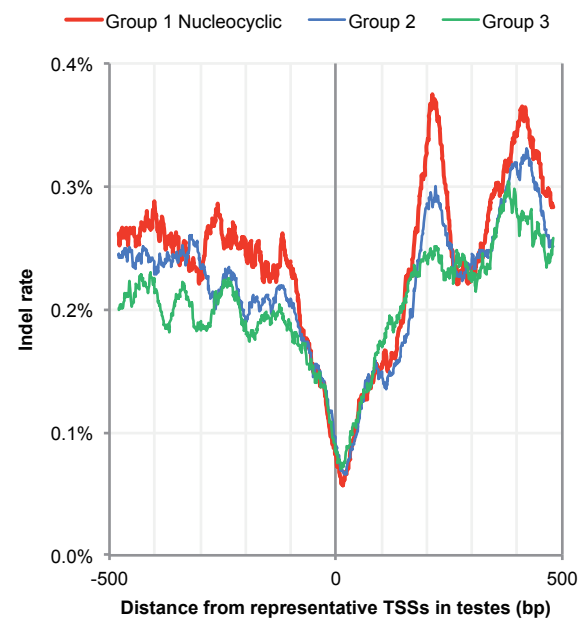

d

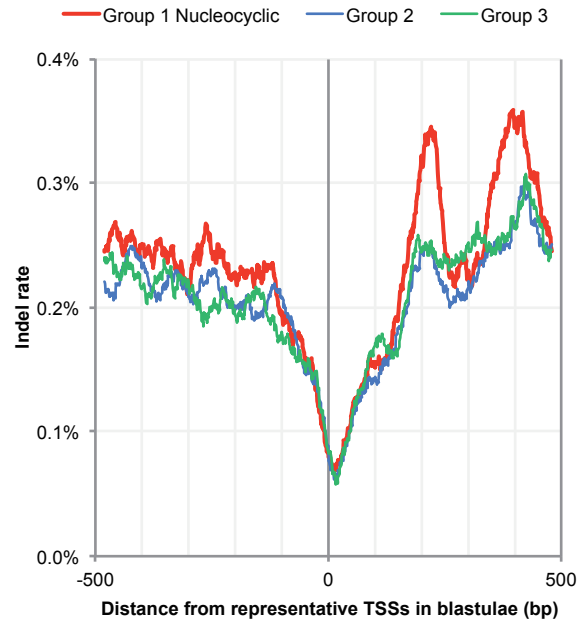

f

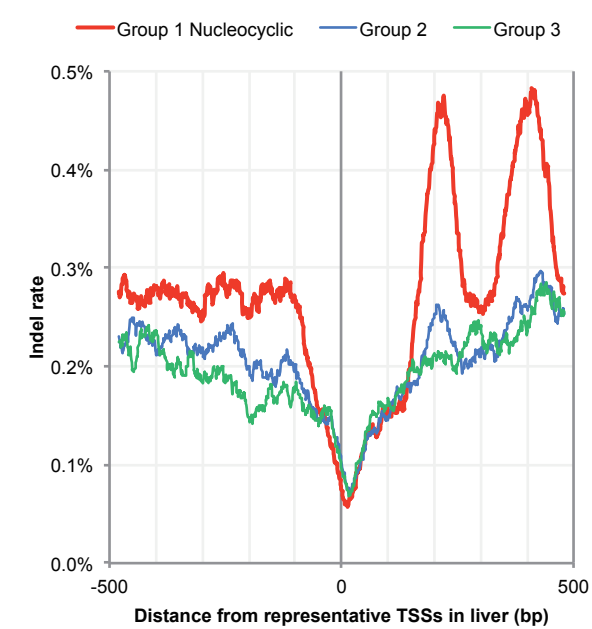

Supplement: Additional file 8: Figure S7. — Single nucleotide mutation rates and indel rates at positions within 500 bp around nucleocyclic/non-nucleocyclic TSSs. a-f. Single nucleotide mutation rates at positions within 500 bp of TSSs in testes (a), blastulae (c) and liver (e), and indel rates at positions within 500 bp of TSSs in testes (b), blastulae (d) and liver (f). A running average over 41-bp window is shown. (PDF 706 kb) [file 12864_2015_2198_MOESM8_ESM.pdf]

a

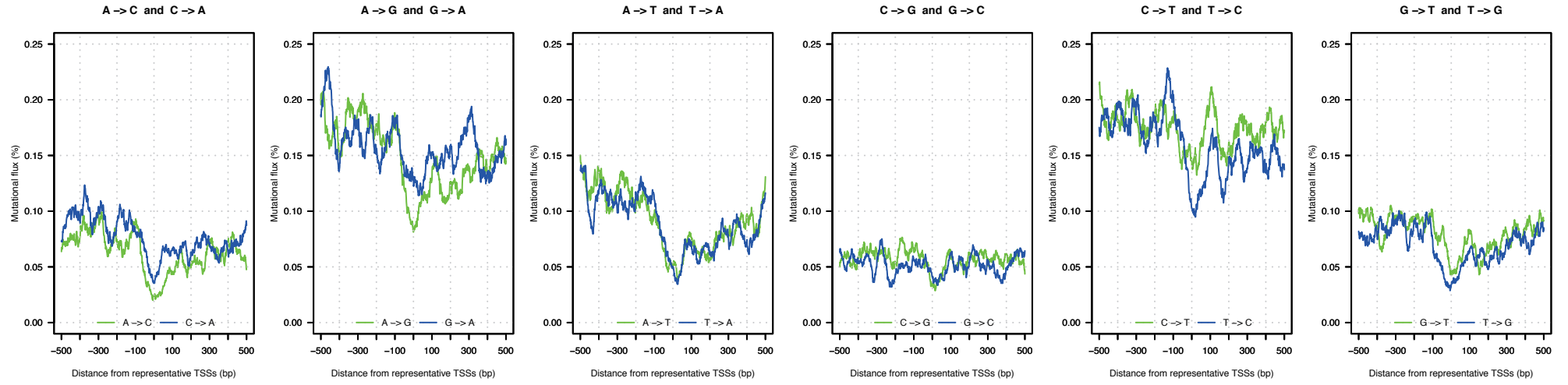

b

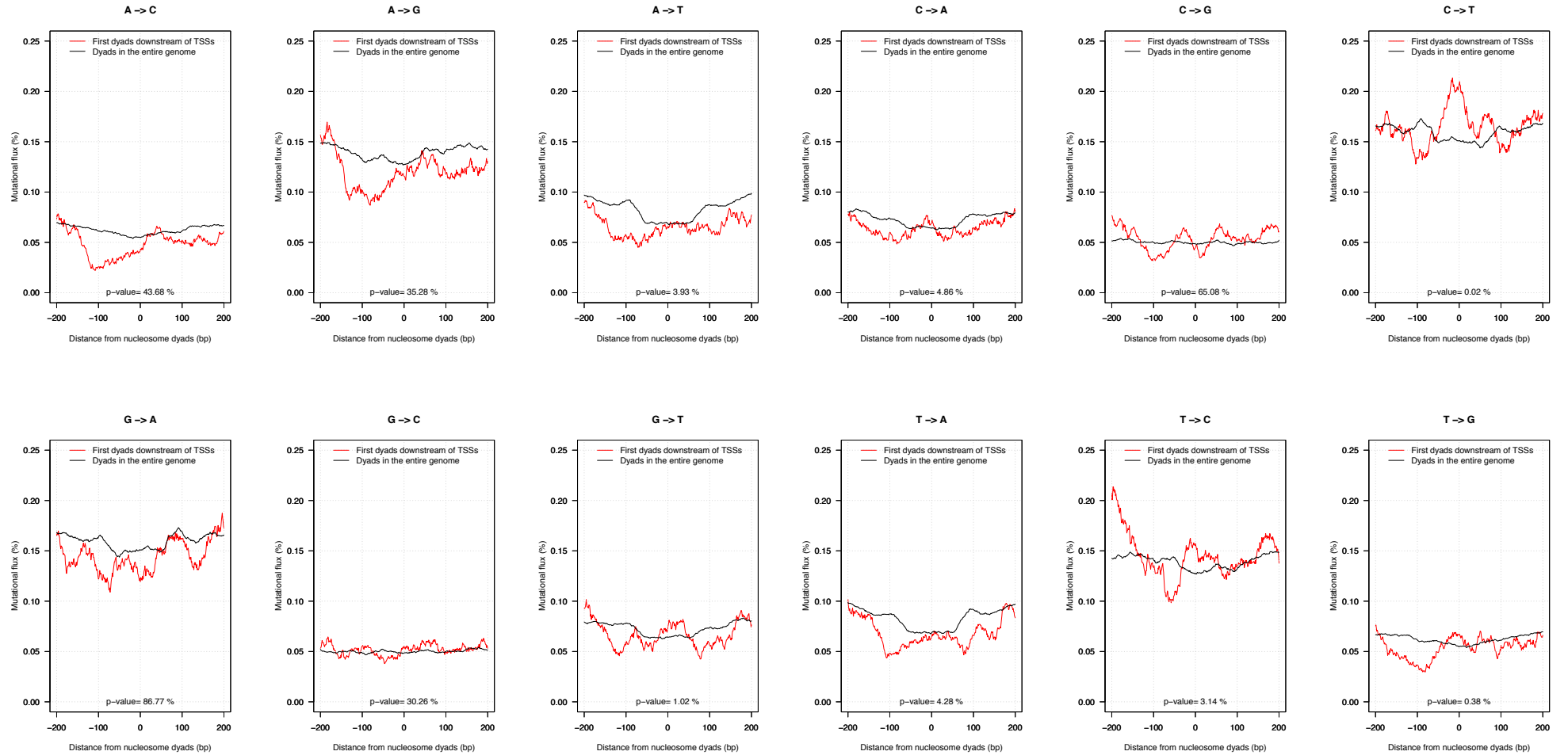

Supplement: Additional file 9: Figure S8. — Atypical evolution around nucleocyclic TSSs in testes. a-b. The definitions of graphs are similar to those in Fig. 3d-e. (PDF 745 kb) [file 12864_2015_2198_MOESM9_ESM.pdf]

a

Testes

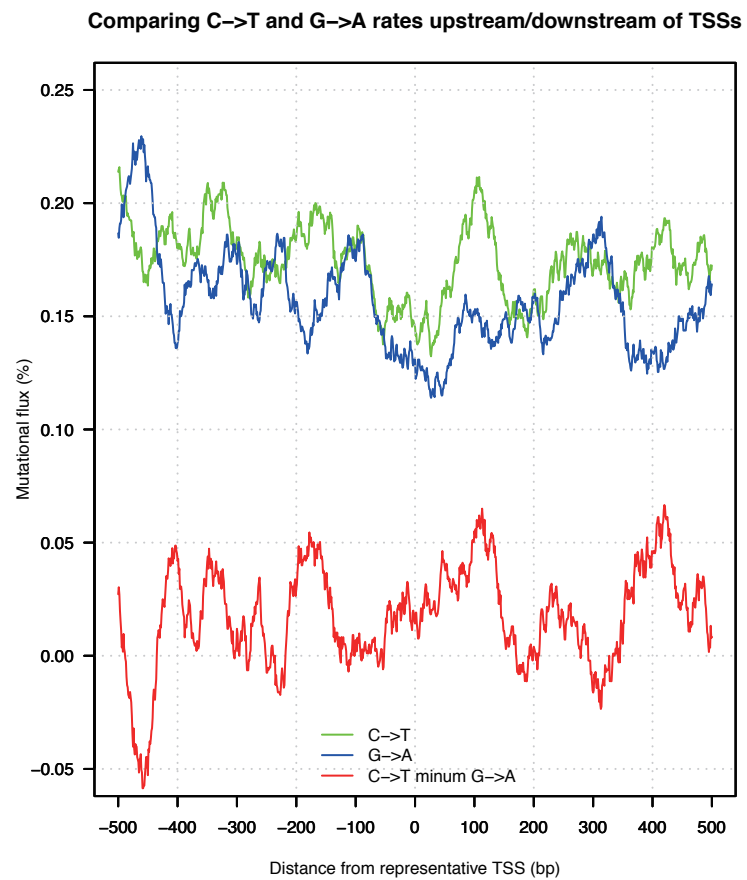

b

Blastulae

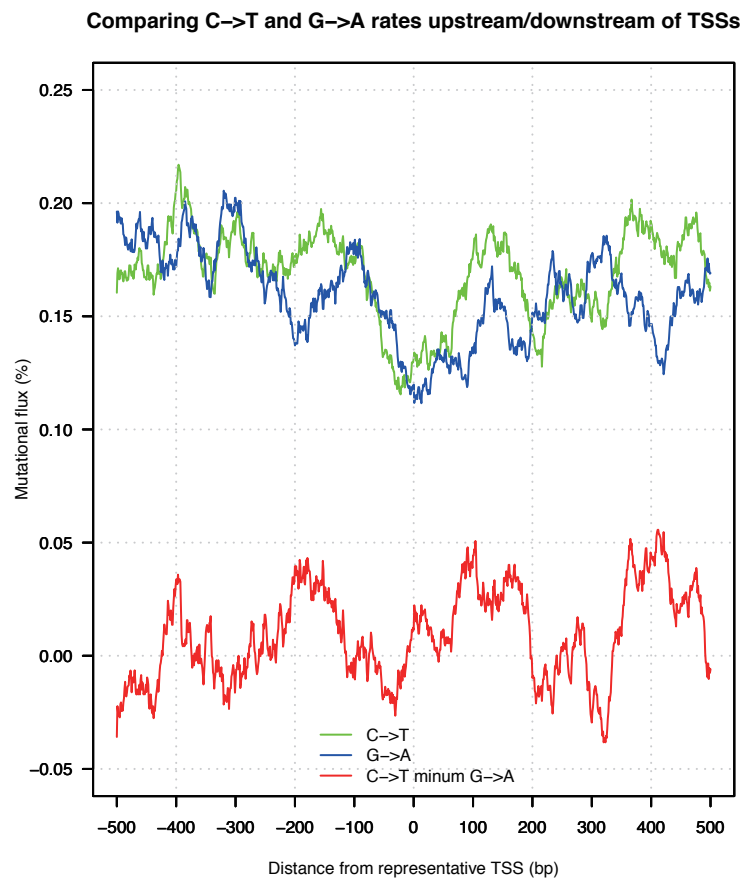

c

Liver

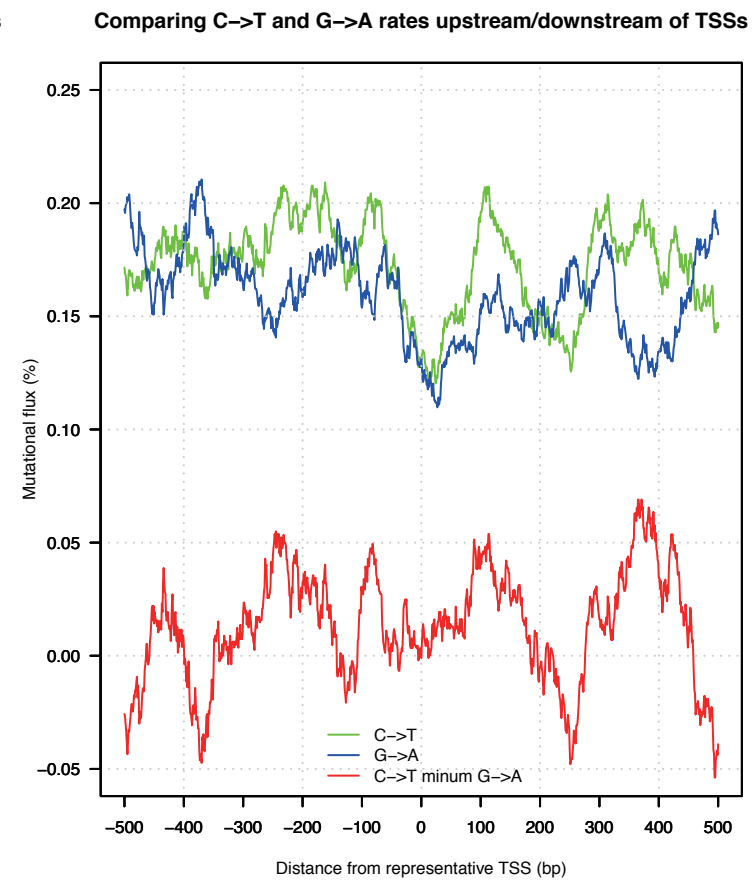

Supplement: Additional file 10: Figure S11. — Possible effect of transcription-coupled repair (TCR). a-c. We observed a significant excess of C to T mutations over G to A mutations in transcribed regions downstream of nucleocyclic TSSs in testes (p = 3.70 % by one-tailed Wilcoxon’s ranksum test described in Methods, a) and in blastulae (p = 4.69 %, b). The p-value for the liver case is 37.92 %. (PDF 629 kb) [file 12864_2015_2198_MOESM10_ESM.pdf]

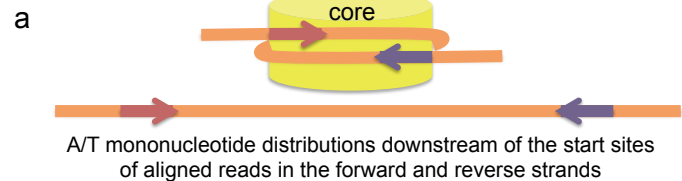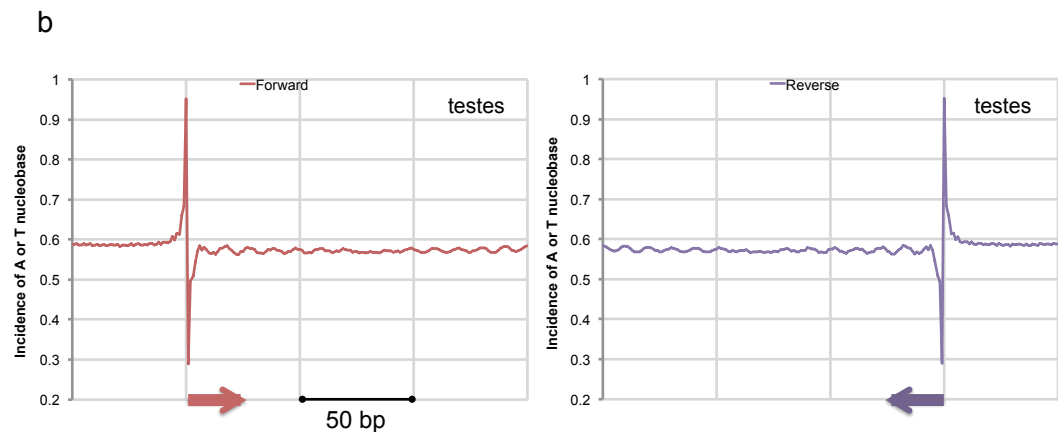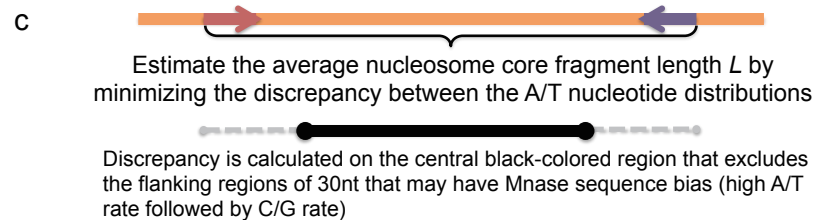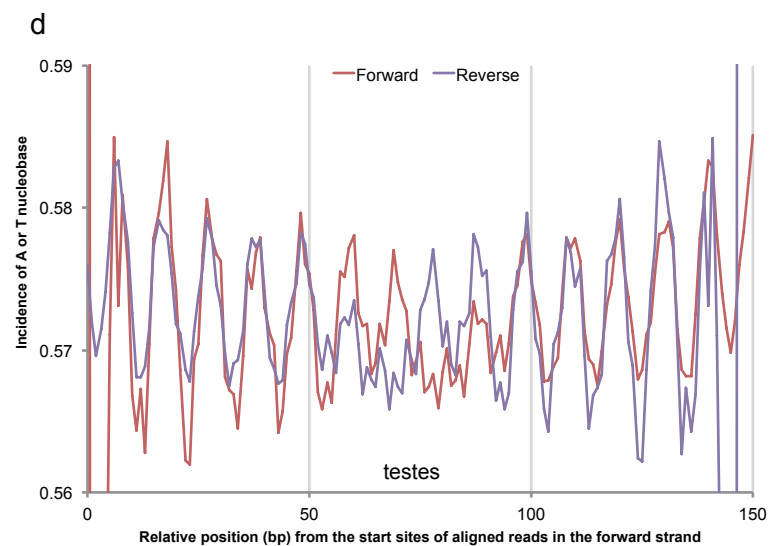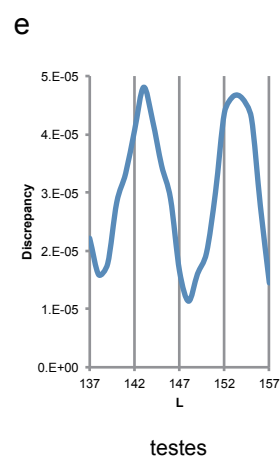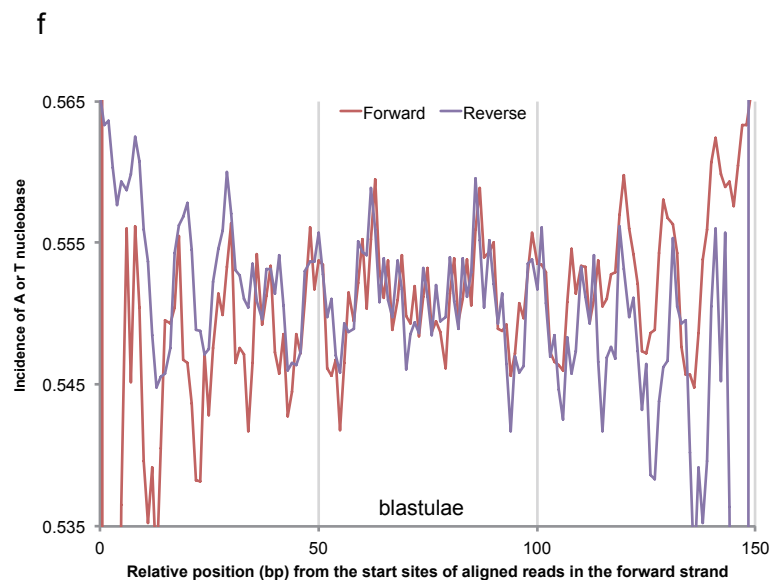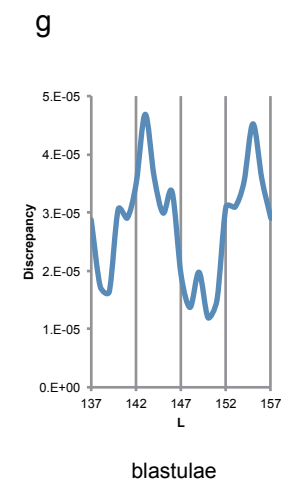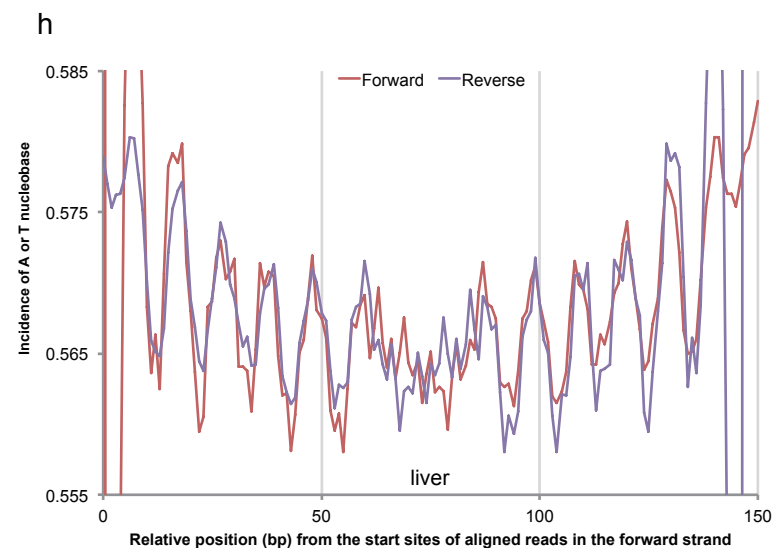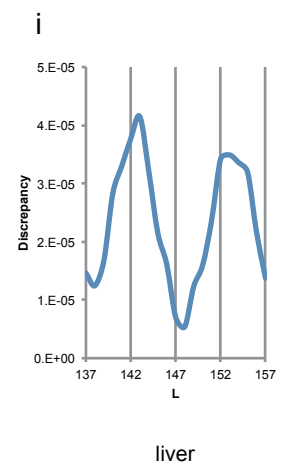

Supplement: Additional file 11: Figure S2. — Estimating the average nucleosome core fragment length L. a. We calculated A/T mononucleotide distributions downstream of the start sites of nucleosome core fragment reads in the forward and reverse strands, separately. b. The A/T distributions of the forward and reverse strands in testes. c-e. We selected the value of L based on a best fit between the independent leading and lagging A/T base composition curves (b) on a genomewide scale, with a concordance in each case between a qualitative matching of the curves (d) and a quantitative “least squares” fit (a minimum discrepancy) between the two (e). Figure d shows the A/T distributions when L is set to the best fit (the minimum discrepancy), 148 bp, as shown in Figure e. f,g. The A/T distributions (f) for the best fit length 150 bp (g) in blastulae. h,i. The A/T distributions (h) for the best fit length 150 bp (i) in liver. (PDF 520 kb) [file 12864_2015_2198_MOESM11_ESM.pdf]
