# Supplementary material for: Associations between nucleosome phasing, sequence asymmetry, and tissue-specific expression in a set of inbred Medaka species
Source: BMC Genomics. 2015 Nov 19;16:978. doi: 10.1186/s12864-015-2198-5 (PMC4653950; doi:10.1186/s12864-015-2198-5)
Supplement: Additional file 7: Figure S10. — Atypical evolution around nucleocyclic TSSs in liver. The definitions of graphs are similar to those in Fig. 3. (PDF 829 kb) [file 12864_2015_2198_MOESM7_ESM.pdf]

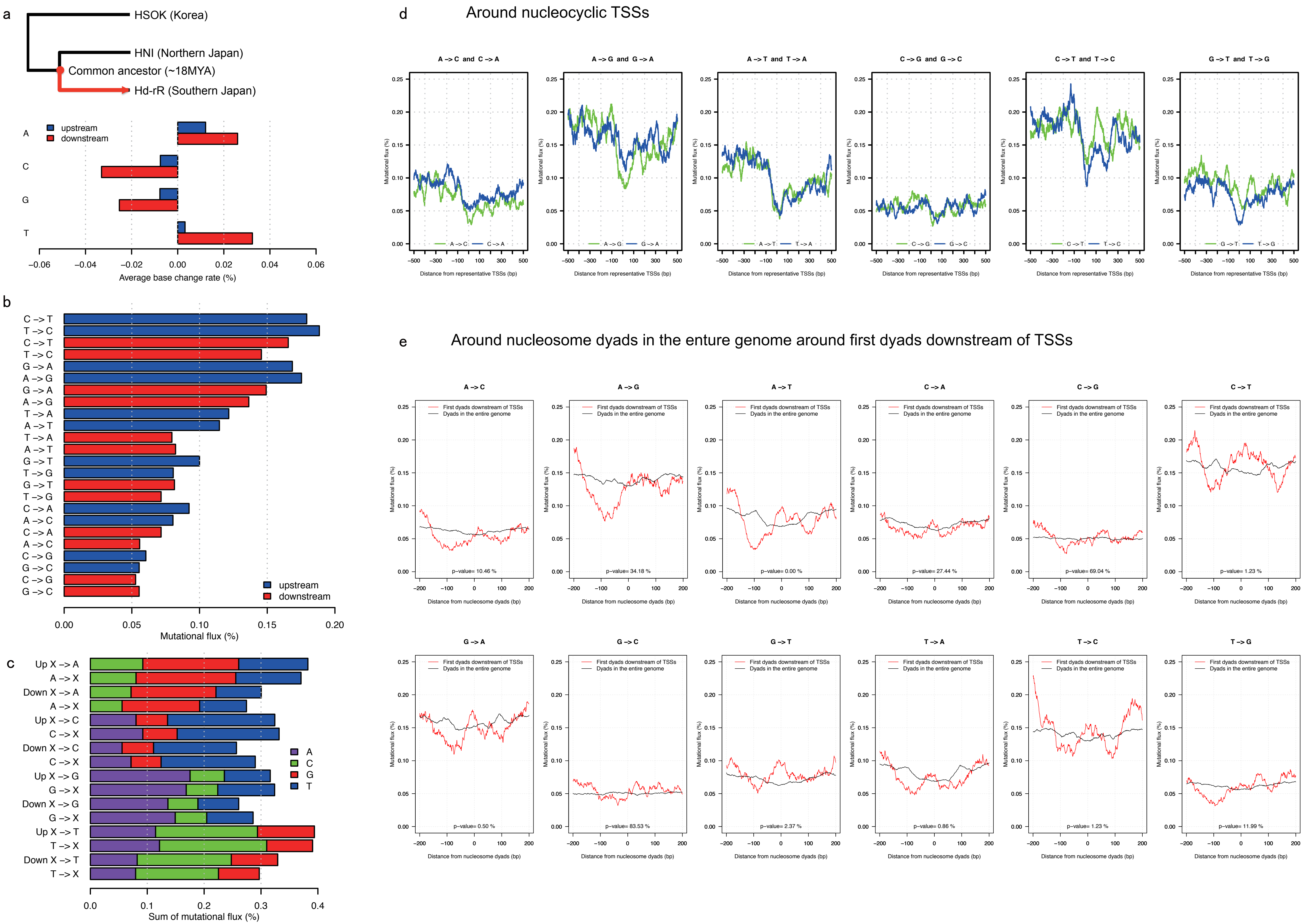
a
HSOK (Korea)
HNI (Northern Japan)
Common ancestor (~18MYA)
Hd-rR (Southern Japan)
A
C
G
T
upstream
downstream
−0.06
−0.04
−0.02
0.00
0.02
0.04
0.06
Average base change rate (%)
b
C −> T
T −> C
G −> A
A −> G
T −> A
A −> T
G −> T
T −> G
C −> A
A −> C
C −> G
G −> C
upstream
downstream
0.00
0.05
0.10
0.15
0.20
Mutational flux (%)
c
Up X −> A
A −> X
Down X −> A
Up X −> C
C −> X
Down X −> C
Up X −> G
G −> X
Down X −> G
Up X −> T
T −> X
Down X −> T
A
C
G
T
0.0
0.1
0.2
0.3
0.4
Sum of mutational flux (%)
d
Around nucleocyclic TSSs
A −> C and C −> A
A −> G and G −> A
A −> T and T −> A
C −> G and G −> C
C −> T and T −> C
G −> T and T −> G
Mutational flux (%)
Distance from representative TSSs (bp)
e
Around nucleosome dyads in the enture genome around first dyads downstream of TSSs
A −> C
A −> G
A −> T
C −> A
C −> G
C −> T
G −> A
G −> C
G −> T
T −> A
T −> C
T −> G
First dyads downstream of TSSs
Dyads in the entire genome
Mutational flux (%)
Distance from nucleosome dyads (bp)
p−value= 10.46 %
p−value= 34.18 %
p−value= 0.00 %
p−value= 27.44 %
p−value= 69.04 %
p−value= 1.23 %
p−value= 0.50 %
p−value= 83.53 %
p−value= 2.37 %
p−value= 0.86 %
p−value= 1.23 %
p−value= 11.99 %
