# Supplementary material for: Associations between nucleosome phasing, sequence asymmetry, and tissue-specific expression in a set of inbred Medaka species
Source: BMC Genomics. 2015 Nov 19;16:978. doi: 10.1186/s12864-015-2198-5 (PMC4653950; doi:10.1186/s12864-015-2198-5)
Supplement: Additional file 6: Figure S9. — Atypical evolution around nucleocyclic TSSs in blastulae. The definitions of graphs are similar to those in Fig. 3. (PDF 835 kb) [file 12864_2015_2198_MOESM6_ESM.pdf]

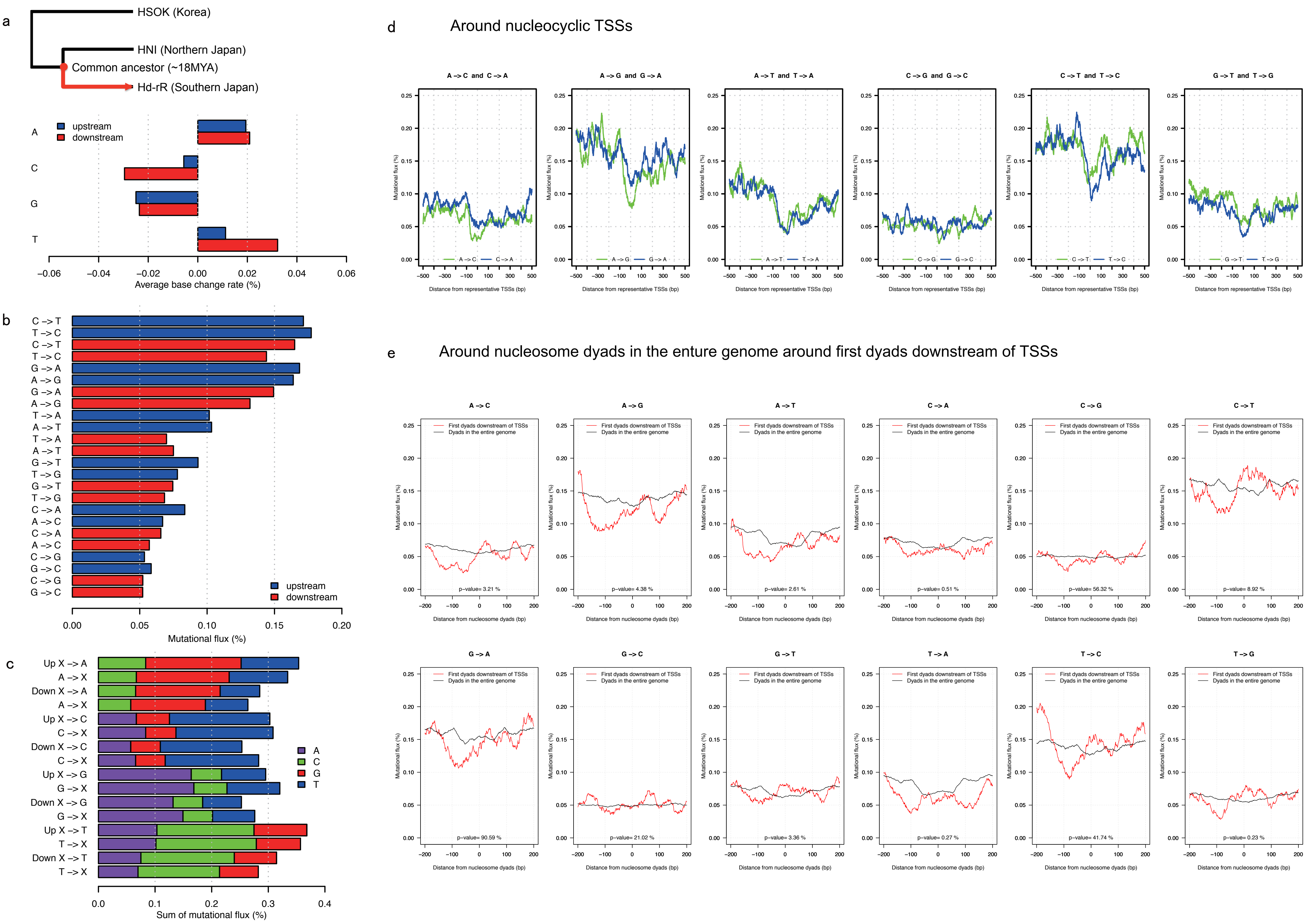
a
HSOK (Korea)
HNI (Northern Japan)
Common ancestor (~18MYA)
Hd-rR (Southern Japan)
upstream
downstream
A
C
G
T
−0.06
−0.04
−0.02
0.00
0.02
0.04
0.06
Average base change rate (%)
b
C –> T
T –> C
G –> A
A –> G
T –> A
A –> T
G –> T
T –> G
C –> A
A –> C
C –> G
G –> C
0.00
0.05
0.10
0.15
0.20
Mutational flux (%)
c
Up X –> A
A –> X
Down X –> A
Up X –> C
C –> X
Down X –> C
Up X –> G
G –> X
Down X –> G
Up X –> T
T –> X
Down X –> T
0.0
0.1
0.2
0.3
0.4
Sum of mutational flux (%)
d
Around nucleocyclic TSSs
A –> C and C –> A
A –> G and G –> A
A –> T and T –> A
C –> G and G –> C
C –> T and T –> C
G –> T and T –> G
Mutational flux (%)
Distance from representative TSSs (bp)
e
Around nucleosome dyads in the enture genome around first dyads downstream of TSSs
A –> C
A –> G
A –> T
C –> A
C –> G
C –> T
G –> A
G –> C
G –> T
T –> A
T –> C
T –> G
First dyads downstream of TSSs
Dyads in the entire genome
p-value= 3.21 %
p-value= 4.38 %
p-value= 2.61 %
p-value= 0.51 %
p-value= 56.32 %
p-value= 8.92 %
p-value= 90.59 %
p-value= 21.02 %
p-value= 3.36 %
p-value= 0.27 %
p-value= 41.74 %
p-value= 0.23 %
Mutational flux (%)
Distance from nucleosome dyads (bp)
